# Integral and peripheral association of proteins and protein complexes with *Yersinia pestis *inner and outer membranes

**DOI:** 10.1186/1477-5956-7-5

**Published:** 2009-02-19

**Authors:** Rembert Pieper, Shih-Ting Huang, David J Clark, Jeffrey M Robinson, Hamid Alami, Prashanth P Parmar, Moo-Jin Suh, Srilatha Kuntumalla, Christine L Bunai, Robert D Perry, Robert D Fleischmann, Scott N Peterson

**Affiliations:** 1 J. Craig Venter Institute, 9704 Medical Center Drive, Rockville, Maryland, USA; 2 Department of Microbiology, Immunology and Molecular Genetics, University of Kentucky, Lexington, Kentucky, USA

## Abstract

*Yersinia pestis *proteins were sequentially extracted from crude membranes with a high salt buffer (2.5 M NaBr), an alkaline solution (180 mM Na_2_CO_3_, pH 11.3) and membrane denaturants (8 M urea, 2 M thiourea and 1% amidosulfobetaine-14). Separation of proteins by 2D gel electrophoresis was followed by identification of more than 600 gene products by MS. Data from differential 2D gel display experiments, comparing protein abundances in cytoplasmic, periplasmic and all three membrane fractions, were used to assign proteins found in the membrane fractions to three protein categories: (i) integral membrane proteins and peripheral membrane proteins with low solubility in aqueous solutions (220 entries); (ii) peripheral membrane proteins with moderate to high solubility in aqueous solutions (127 entries); (iii) cytoplasmic or ribosomal membrane-contaminating proteins (80 entries). Thirty-one proteins were experimentally associated with the outer membrane (OM). *Circa *50 proteins thought to be part of membrane-localized, multi-subunit complexes were identified in high M_r _fractions of membrane extracts via size exclusion chromatography. This data supported biologically meaningful assignments of many proteins to the membrane periphery. Since only 32 inner membrane (IM) proteins with two or more predicted transmembrane domains (TMDs) were profiled in 2D gels, we resorted to a proteomic analysis by 2D-LC-MS/MS. Ninety-four additional IM proteins with two or more TMDs were identified. The total number of proteins associated with *Y. pestis *membranes increased to 456 and included representatives of all six *β*-barrel OM protein families and 25 distinct IM transporter families.

## Background

*Yersinia pestis*, a Gram-negative bacterium, is the causative agent of the bubonic and pneumonic plague. The pathogenic lifestyle of this microbe involves two distinct life stages, one in the flea vector, the other in mammalian hosts, primarily rodents [1]. Humans are a dead-end host and not part of the flea-mammal cycle. *Y. pestis *strains associated with high virulence have been divided into three classical biovars (antiqua, mediaevalis and orientalis) based on differences in their abilities to ferment glycerol and reduce nitrate. A fourth biovar (microtus) has been proposed on the basis of low virulence and reduced transmission [2-4]. Complete DNA sequence data exist for the genomes of each of these four biovars [5-8]. The gene organization and complete DNA sequences of three *Y. pestis *virulence-associated plasmids were also determined [9,10]. The pCD1 plasmid, shared with other human pathogenic *Yersinia *species, encodes a suite of proteins required for a functional type III secretion system (T3SS) and host infection. A temperature increase from 26–30 to 37°C and host cell contact or a low Ca^2+ ^concentration induce expression of these proteins [11]. Most *Y. pestis *strains harbor two unique plasmids, pPCP1 and pMT1, not present in *Y. pseudotuberculosis*. These plasmids encode factors such as the plasminogen activator protease (Pla), required for mammalian pathogenesis [12], the *Yersinia *murine toxin (Ymt), required for colonization of the mid-gut of fleas [13,14], and the F1 capsular antigen (Caf1) [15]. The F1 antigen causes *in vitro *resistance to phagocytosis, but its role in mammalian virulence is unclear [16]. In addition, the genetically unstable chromosomal 102-kb *pgm *locus is important for full virulence of the bubonic plague in mammals and for transmission via blocked fleas [17,18]. It encodes the yersiniabactin siderophore-dependent iron transport (Ybt) system [19,20] and the Hms-dependent biofilm system. Biofilm formation allows colonization of the flea proventriculus, causing blockage which induces active feeding behaviour [21,22]. Gene expression and proteomic studies have demonstrated that numerous plasmid- and *pgm *locus-encoded genes are differentially expressed at 26–30 *vs*. 37°C [23-26] and during temperature transition [27]. Some of these changes are in agreement with specific functional roles of the encoded proteins in one of the two life stages [24,25,27].

Several of the characterized *Y. pestis *virulence factors, including effector proteins and their transporters, are membrane-associated. Global profiling of *Y. pestis *membrane compartments holds promise for the discovery of novel virulence- and life stage-associated proteins. A proteomic effort to designate life stage-specific *Y. pestis *KIM protein biomarkers included several membrane-associated proteins [24]. The latter study did not focus on determining membrane protein localizations, and a comprehensive *Y. pestis *membrane proteome survey has not been published to date. The assessment of membrane association of proteins annotated in *Y. pestis *genome databases has largely relied on sequence similarities to orthologous proteins in *Escherichia coli *and other Gram-negative bacteria or on predictions of conserved membrane integration motifs. Extensive membrane proteome surveys have been reported for *E. coli *[28,29], *Pseudomonas aeruginosa *[30] and *Rhodobacter sphaeroides *[31]. Strategies for membrane proteome analysis using non-recombinant methodologies are diverse and were presented in review articles [32,33]. High salt buffers and alkaline Na_2_CO_3 _solutions are often used to solubilize peripheral membrane proteins. To dentaure membranes and solubilize integral membrane proteins, detergents such as CHAPS, laurylsarcosine and amidosulfobetaine-14 (ASB-14) are frequently used in combination with chaotropic reagents, 7–9 M urea, for instance. With 2D gels, it is most challenging to profile low abundance proteins harboring several transmembrane domains (TMDs). A novel method was recently described to increase the depth of integral membrane protein surveys [34]. Proteolytic digestion of bacterial membrane fractions in organic solvents resulted in the enrichment of TMD proteins, which were identified with increased sequence coverage by 2D-LC-MS/MS.

Here, the *Y. pestis *membrane proteome derived from stationary phase cells grown at 26°C was investigated primarily. Both 2D gel- and 2D-LC-MS/MS-based approaches were used to separate and identify more than 500 proteins apparently associated with the inner membrane (IM) or the outer membrane (OM) of this pathogen. Other methodologies were also employed to substantiate the experimental findings. Algorithms such as TMHMM, LipoP and BOMP and the subcellular localization tool PSORTb [35] were used to recognize various conserved membrane association motifs. Additional evidence for membrane association of proteins can be obtained from co-fractionation experiments, *e.g*. size exclusion chromatography (SEC) or non-denaturing BN-PAGE [36], because membrane proteins are often part of multi-subunit protein complexes. Thus, we included a protein survey of high M_r _membrane-derived SEC fractions. Information gleaned from data in this report is useful to improve protein annotations in *Y. pestis *genome databases and to select targets for functional and protein-protein interaction studies.

## Methods

### Materials

Specialty reagents used for subcellular fractionation, 2D gel and MS were previously described [37]. The detergents Triton X-100, CHAPS and ASB-14 were purchased from Calbiochem (LaJolla, CA). RNase and DNase I (each from bovine pancreas) were from Sigma-Aldrich (St. Louis, MO). Sequencing grade porcine trypsin and chymotrypsin were from Promega (Madison, WI) and Roche (Indianapolis, IN), respectively.

### Bacterial strains and culture conditions

The *Y. pestis *KIM6+ strain used in this study is an avirulent derivative of the fully virulent KIM strain, which was cured of the pCD1 plasmid. The chromosomal *pgm *locus and the plasmids pMT1 and pPCP1 are present in KIM6+ [38]. Cells were maintained and grown in chemically defined media (PMH2) as previously described [39]. Aliquots of KIM6+ stock solutions in 33 mM K_2_HPO_4 _(pH 7.5) were used to inoculate 5–10 mL pre-cultures in PMH2. Pre-cultures were transferred to shaker flasks with 0.25–1.0 L PMH2 and grown overnight, at 26 and 37°C, to an OD_600 _of 1.9–2.4. To limit phosphate (P_i_) concentrations during cell growth, 0.12 mM K_2_HPO_4 _(1/20^th ^of the normal concentration) was added to PMH2. Cells in P_i_-deficient media were grown overnight to an OD_600 _of 1.0–1.4. Bacterial cells were harvested by centrifugation at 8,000 × *g *for 15 min at 4°C and washed with a 30-fold volume of 33 mM K_2_HPO_4 _(pH 7.5).

### Preparation of *Y. pestis *cell lysates and subcellular fractionations

Whole cell lysate (WCL) supernatants were only prepared from cells grown to stationary phase at 26°C. Cells were resuspended in 25 mM Tris-OAc (pH 7.8), 5 mM EDTA, 150 *μ*g/ml lysozyme, 2 mM PMSF, 0.05% Triton X-100 and 1 mM benzamidine. After incubation for 30 min at 20°C and intermittent vortexing, the cell lysate was centrifuged at 208,000 × *g *for 90 min at 4°C. The pellet was discarded, while the supernatant was recovered and concentrated to *ca*. 2–5 mg/mL protein for analysis in 2D gels. To perform subcellular fractionation experiments, a lysozyme/EDTA spheroplasting method previously described [37,40] was applied. Periplasmic supernatant (PPS) fractions were exchanged into buffer A (25 mM NaHCO_3_, pH 7.8, 1 mM EDTA and 1 mM benzamidine) and concentrated in Ultrafree-4 filter units (NMWL 10,000; Fisher Scientific) to *ca*. 2–5 mg/mL protein for analysis in 2D gels. Diluted protein fractions described in the following paragraphs were concentrated accordingly, unless otherwise stated.

Spheroplast pellets were subjected to lysis in an ice-cold solution of 0.25 M sucrose, 10 mM Tris-OAc (pH 7.8), 5 mM EDTA, 0.2 mM DTT, and protease inhibitors (1 mM benzamidine, 10 *μ*g/ml leupeptin, 5 *μ*g/ml pepstatin, 10 *μ*g/ml N_*α*_-p-Tosyl-L-arginine methyl ester and 2 mM PMSF), using *ca*. 7–10 mL/g wet cell pellet weight. Homogenized spheroplasts were sonicated on ice for 3 min in 10 sec on/off cycles at amplitude 30 (Branson sonicator), followed by incubation with 10 mM MgCl_2_, DNase I (10 *μ*g/ml) and RNase (10 *μ*g/ml) for 1 h at 20°C. Thereafter, 150 mM NaCl was added to the lysate, which was left on ice for 30 min and then centrifuged at 208,000 × *g *for 1 h at 4°C. The cytoplasmic supernatant (CYP) fraction was concentrated to *ca*. 2–5 mg/mL protein for analysis in 2D gels. The pellet contained crude membranes and could be frozen at -80°C. To isolate OM fractions, the unfrozen membrane pellet was resuspended in 15% sucrose, 50 mM Tris-OAc, 2 mM Na-EDTA and 0.05% Triton X-100. Discontinuous sucrose density gradient centrifugation for 17 h was performed as previously described [41]. The OM-enriched fraction banded as a white layer with a density of *ca*. 1.25 g/mL and, after dilution with 50 mM Tris-OAc and 2 mM EDTA, was centrifuged again at 208,000 × *g *for 2 h.

### Stepwise extraction of proteins from *Y. pestis *membrane preparations

Crude membrane pellets were resuspended in a *ca*. 15-fold volume of 10 mM Tris-HCl (pH 7.8), 5 mM EDTA, 0.2 mM DTT, 10 *μ*g/ml Leupeptin, 5 *μ*g/ml Pepstatin, 10 *μ*g/ml N_*α*_-p-Tosyl-L-arginine methyl ester and 2 mM PMSF. Sodium bromide was added at a 2.5 M concentration. The suspension was stirred for 1 h at 20°C and centrifuged at 50,000 × *g *for 1 h at 4°C. The high salt-extracted supernatant (hs-MBR) fraction was desalted and concentrated to *ca*. 1–2 mg/mL protein for analysis in 2D gels. The remaining membrane pellet was re-homogenized in ice-cold 0.18 M Na_2_CO_3 _(pH 11.3), 50 mM DTT, 1 mM CaCl_2_, 1 mM MgCl_2 _and 1 mM MnCl. The suspension was stirred for 1 h at 4°C, followed by centrifugation at 50,000 × *g *for 1 h at 4°C. The supernatant of the high pH extraction step (hpH-MBR fraction) was concentrated to *ca*. 1–2 mg/mL protein. The insoluble membrane pellet was frozen at -80°C or immediately resuspended in 8 M urea, 2 M thiourea, 1% (w/v) ASB-14, 2 mM tributylphosphine and 0.5% (v/v) Bio-Lyte^® ^pH 3–10 carrier ampholytes. Protein solubilization for 30 min at 20°C in the denaturing buffer was followed by centrifugation at 16,100 × *g *for 15 min. The residual pellet was discarded. The protein concentration of the urea/detergent-extracted supernatant (usb-MBR) fraction was estimated from the Coomassie brilliant blue G-250 (CBB)-staining intensity in SDS-PAGE gels. Finally, to extract proteins peripherally associated with the OM/cell surface, stationary phase KIM6+ cells were resuspended in 0.25 M sucrose, 25 mM Tris-OAc (pH 7.8), 2 mM PMSF and 1 M NaCl (10 mL/g cell weight) and agitated gently for 30 min. Following centrifugation at 8,000 × *g *for 15 min, the high salt-extracted cell surface supernatant (hs-CS) fraction was recovered and filtered through a 0.45 *μ*m pore size filter. Extracted proteins were concentrated in buffer A and lyophilized prior to analysis in 2D gels.

### Size exclusion chromatography

Triton X-100 (0.075% w/v) was added to a 1 mg protein aliquot of the hpH-MBR fraction derived from stationary phase *Y. pestis *cells grown at 26°C. The solubilized sample (*ca*. 1 mL) was applied to a Superdex™ 200 column (1.6 × 100 cm; GE Healthcare) equilibrated in 100 mM Na_2_HPO_4 _(pH 7.5), 150 mM NaCl, 2 mM BAM, 2 mM EDTA and 0.05% (w/v) Triton X-100. At a flow rate of 0.75 mL/min, proteins were fractionated at 20°C. To calibrate the column (elution time *vs*. M_r_), 0.5 mg of a high M_r _protein standard mix including thyreoglobulin (670 kDa), bovine IgG (158 kDa), ovalbumin (45 kDa), myoglobin (17 kDa) and vitamin B12 (1.4 kDa) was fractionated via SEC. Protein elutions were monitored at a wavelength of A_280_. Fractions were collected from the void volume (~500 kDa) to an elution time corresponding to a protein M_r _of *ca*. 20 kDa, concentrated in buffer A and lyophilized for analysis in 2D gels.

### Protein analysis in 2D gels

Protein concentrations in soluble fractions were measured using the BCA assay (Sigma-Alrich, St. Louis, MO). Samples containing 75–150 *μ*g protein were diluted with 8 M urea, 2 M thiourea, 4% (w/v) CHAPS, 18 mM DTT and 0.5% (v/v) Bio-Lyte^® ^pH 3–10 carrier ampholytes to 400–450 *μ*L for IPG gel rehydration loading or 100–150 *μ*L for IPG gel anodic cup loading. The usb-MBR fractions derived from either mixed membranes or OM-enriched preparations were applied to IPG gels in the aforementioned denaturing urea/ASB-14 solubilization solution. Electrophoretic protein separation in 1^st ^dimension 24 cm IPG strips (pH ranges 4–7 and 3–10) and 2^nd ^dimension SDS-PAGE slab gels (25 × 19.5 × 0.15 cm) as well as gel staining with the dyes CBB and Sypro^® ^Ruby and gel image processing into 16-bit TIFF files were carried out as previously described [42]. Spot positions of 25 cytoplasmic proteins were used as landmarks for M_r _and pI calibrations in 2D gels.

### Differential display analysis to assess protein enrichment in membrane fractions

First, semi-quantitative differential gel display experiments were performed. PPS, CYP, hs-CS, hs-MBR, hpH-MBR and usb-MBR fractions (two to four gels each) were subjected to spot matching, which was confirmed by MS data, and differential spot quantitation. The software used for 2D gel image analysis was Proteomweaver *vs*.4.0 (Bio-Rad, Hercules, CA). The analysis mode included pre-match normalization, as previously reported [37]. Post-match normalization was not performed, and P-values were not determined, because low spot pattern similarity among the fractions confounded such analysis steps. This semi-quantitative analysis was comprehensive for 2D gels (in the pH ranges 4–7 and 7–10) representing PPS, CYP, hs-MBR, hpH-MBR and usb-MBR fractions of KIM6+ cells grown to stationary phase at 26°C. Data for a small subset of proteins much more abundant in equivalent fractions of cells either grown in P_i_-limited media at 26°C or to stationary phase at 37°C were derived from 2D gels (in the pH range 4–7) representing these two growth conditions. The densitometric intensity value of a spot in a given fraction was usually averaged because spots were usually detected in more than one gel per fraction. This data is presented in the columns O-R of the Supplemental Table (Additional File 1). Due to the small number of gels and lack of biological replicates, coefficients of variation (CVs) were not determined.

Secondly, a more quantitative differential display experiment affording assessments of the reproducibility of protein enrichments in distinct subcellular fractions was performed. All data were derived from 2D gels in the pH range 4–7, representing CYP, hs-MBR, hpH-MBR and usb-MBR fractions of KIM6+ cells grown to stationary phase (in complete PMH2) at 26°C. Here, each subcellular fraction (group) was represented by three separate cell culture batches and at least six gels per group. For a protein forming a spot train in a given fraction, spot quantities per train were summed. Geometric means (X_N_) and CVs were determined and are presented in the columns I-L of the Supplemental Table. If well-resolved spots were not detected for a given protein, X_N _and CV values were not obtained. All of the data were used to calculate enrichment factors of proteins in membrane fractions, specifically E_M_, the ratio of a protein quantity in all membrane fractions *vs*. the CYP fraction, and E_IM_, the ratio of a protein quantity in the hpH-MBR and usb-MBR fractions *vs*. the hs-MBR fraction (columns denoted E_M _and E_IM_, Table 1; Additional File 2). The equations used to calculate these factors were E_M _= (X_N_)^1/N^_hs-MBR _× *μ*_TP _× *μ*_GP_^-1 ^+ (X_N_)^1/N^_hpH-MBR _× *μ*_TP _× *μ*_GP_^-1 ^+ (X_N_)^1/N^_usb-MBR _× *μ*_TP _× *μ*_GP_^-1^/(X_N_)^1/N^_CYP _× *μ*_TP _× *μ*_GP_^-1 ^and E_IM _= (X_N_)^1/N^_hpH-MBR _× *μ*_TP _× *μ*_GP_^-1 ^+ (X_N_)^1/N^_usb-MBR _× *μ*_TP _× *μ*_GP_^-1^/(X_N_)^1/N^_hs-MBR _× *μ*_TP _× *μ*_GP_^-1^, respectively, where X_N _is the geometric mean of a protein quantity, *μ*_GP _is the average quantity of gel-loaded protein and *μ*_TP _is the average quantity of total protein for a given fraction normalized based on wet cell pellet weight.

### Mass spectrometry analysis

Methods for spot picking and peptide digestion were previously described [42]. Peptide digests were analyzed using a MALDI-TOFTOF mass spectrometer (4700 Proteomics Analyzer, Applied Biosystems, Framingham, MA) and a nano-electrospray LC-MS/MS system (LTQ-IT mass spectrometer, Thermo-Finnigan, San Jose, CA) equipped with an Agilent 1100 series solvent delivery system (Agilent, Palo Alto, CA). Peptide separation for LC-MS/MS analysis was performed using a PicoTip microcapillary reversed-phase column (BioBasic C_18_, 75 *μ*m × 10 cm, New Objective, Woburn, MA) at a flow rate of 0.35 *μ*L/min. Technical aspects of the MS analysis were previously described [42]. MS and MS/MS data were searched against the latest release of the *Y. pestis *KIM strain subset of the NCBInr database using the Mascot searching engine vs.2.2 (Matrix Science, London, UK). Carbamidomethyl was invariably selected as a fixed modification. One missed tryptic cleavage was allowed. MALDI search parameters (+1 ions) included mass error tolerances of ± 100 ppm for peptide ions and ± 0.2 Da for fragment ions. LTQ-IT search parameters (+1, +2 and +3 ions) included mass error tolerances of ± 1.4 Da for peptide ions and ± 0.5 Da for fragment ions. Protein identifications were accepted as significant when a Mascot protein score >75 and at least one peptide e-value <0.1 were reported. To accept a Mascot score between 50 and 75, a protein had to be identified at least three times with at least one peptide e-value <0.1 each. Using the automatic decoy database search option in Mascot with a default significance threshold of 0.05, the peptide false discovery rate was *ca*. 6%. Since at least two peptides were identified in *ca*. 95% of all LC-MS/MS analyses per spot, the final protein false discovery rate was estimated to be lower than 0.3%.

### Membrane protein profiling by 2D-LC-MS/MS

To analyze proteins via 2D-LC-MS/MS, a recently described method [34] was employed. Briefly, *ca*. 200 *μ*g of a crude membrane pellet isolated from *Y. pestis *KIM6+ cells grown at 26°C in P_i_-rich media was predigested with trypsin (20:1 w/w) in 25 mM NH_4_HCO_3 _(pH 8.5) overnight at 37°C. The sample was pelleted by centrifugation at 100,000 × *g*, washed twice with an ice-cold 0.1 M Na_2_CO_3 _solution and water, and resuspended in methanol/25 mM NH_4_HCO_3_, pH 8.5 (60:40, v/v). After sonication at an amplitude 5 for 10 min and overnight digestion with trypsin/chymotrypsin (1:100 each, w/w), the sample was centrifuged at 100,000 × *g*, lyophilized and adjusted with AcOH to pH 3.0 for peptide separation via strong cation exchange chromatography (SCX). A 50 × 4.6 mm Polysulfoethyl A column (PolyLC Inc, Columbia, MD) was equilibrated with 5 mM KH_2_PO_4 _(pH 3) in water/AcCN (75:25). At a flow rate of 1 ml/min, a linear salt gradient elution to 0.35 mM KCl with 5 mM KH_2_PO_4 _(pH 3) in water/AcCN (75:25) was performed. More than 20 fractions were collected, lyophilized, re-suspended in 5% formic acid and applied to LC-MS/MS on the LTQ system, as described in the previous paragraph. A full range mass-scan (100–1500 m/z) was followed by top five data-dependent MS/MS scans. The digestion step resulted in amino acid cleavages C-terminal to K, R, W, Y, F and L residues. Criteria for protein identification using Mascot included two missed cleavages [34]. A minimum of two MS/MS peptide identifications per protein were required. When Mascot scores were between 50 and 75, a protein had to be identified in at least two separate analyses, with at least one peptide e-value <0.1 in each case. To determine the false positive rate, a search with a decoy database composed of a shuffled *Y. pestis *KIM sequence database was performed. Using these criteria, the false positive rate was estimated to be lower than 2%.

### Bioinformatic analysis tools

To predict motifs for lipoproteins, TMDs and export signal sequences in *Y. pestis *proteins, queries for the entire *Y. pestis *KIM strain database [43] were performed. The algorithms used were LipoP, SignalP, TatP and TMHMM, all of which were accessed at [44]. *In silico *predictions of subcellular localizations were obtained from PSORTb vs.2.0 searches [35]. The algorithm BOMP [45] was used to search for *β*-barrel OM protein motifs. To assign proteins to membrane transporter categories, the *Y. pestis *KIM strain subset of the database TransportDB was queried [46].

## Results

### Experimental approaches to assess protein association with *Y. pestis *membranes

**Figure 1:**
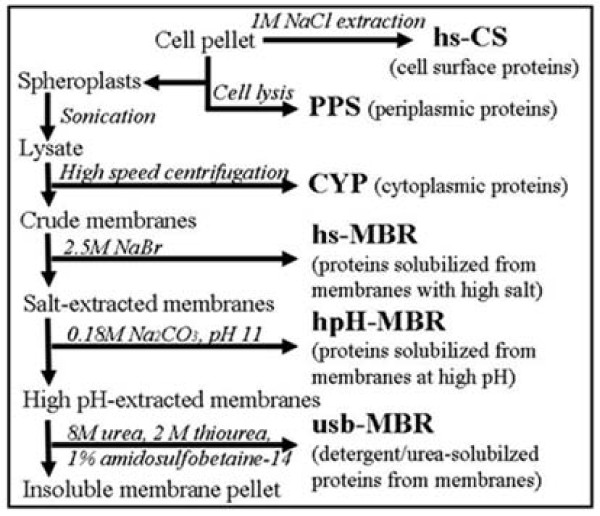
**Subcellular fractionation and stepwise protein extraction from *Y. pestis *KIM6+ membrane fractions**.

As illustrated in Figure 1, six subcellular fractions were isolated from *Y. pestis *KIM6+ cell lysates. In addition to a cell surface (hs-CS) fraction, a periplasmic (PPS) fraction was recovered. Spheroplasts were lysed, and soluble proteins of the cytoplasm (CYP fraction) were isolated. Crude membrane pellets were extracted sequentially, first with 2.5 M NaBr (hs-MBR fraction), then with Na_2_CO_3 _at pH 11.3 (hpH-MBR fraction), and finally with 8 M urea, 2 M thiourea and 1% ASB-14 (usb-MBR fraction). Fractions derived from cells grown to stationary phase at 26°C were subjected to extensive analyses via differential 2D gel display, while the corresponding analyses for two other growth conditions (P_i_-limited media at 26°C; stationary phase at 37°C) were limited to a few proteins specifically expressed under such growth conditions. Detailed descriptions for the analysis modes are provided in the Methods. The inclusion of CYP and PPS fractions allowed us to discriminate between true peripheral membrane proteins and contamination of membrane fractions with 'non-specifically binding' soluble proteins or protein aggregates. Non-specific binding of proteins to membranes is caused by physicochemical changes in cells, resulting in partial protein denaturation and exposure of hydrophobic surfaces normally hidden in the proteins' interior. Abnormal protein aggregates form *in vivo *and, more extensively and especially after cell lysis, *in vitro*; these aggregates co-fractionate with membranes [47].

**Figure 2:**
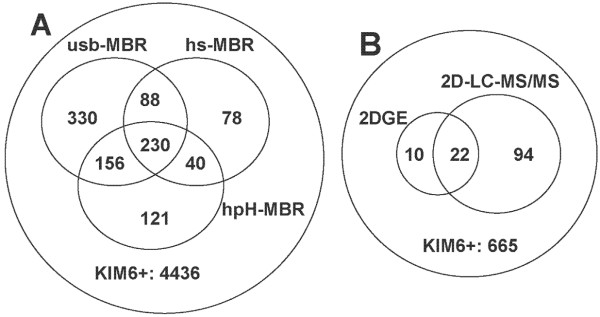
**Venn diagrams of proteins identified from 2D gels derived from three different membrane fractions (A) and proteins with more than one transmembrane domain (TMD) identified in 2D gel *vs*. 2D-LC-MS/MS experiments (B)**. Acronyms are described in the flowchart of Figure 1. 2D gel-based protein identifications included several *Y. pestis *growth conditions, while those identified from 2D-LC-MS/MS efforts were limited to stationary phase cell growth at 26°C. The analysis of TMDs is based on bioinformatic motif predictions by the algorithm TMHMM.

Due to the above-mentioned causes, overlapping protein identifications among CYP, PPS and all three membrane fractions were not unexpected. Less than 50% of more than 600 distinct proteins were observed only in the membrane fractions. As shown in the Venn diagram of Figure 2A, protein overlaps among the membrane fractions were also extensive and indicative of a gradual protein extraction process from membranes. Indeed, quite a few relatively abundant proteins known to bind to membranes in different modes (*e.g*. OmpA, ManX and ProV) were detected in hs-MBR, hpH-MBR and usb-MBR fractions. Therefore, comparative protein abundance measurements in 2D gels were more insightful than the 'observation' or 'non-observation' of a protein in a given subcellular fraction. Such data were available for 421 proteins using semi-quantitative differential 2D spot abundance measurements (columns O-R, Additional File 1) and for 280 of 421 proteins using quantitative measurements including biological replicates and CVs (columns I-L, Additional File 1). The latter dataset allowed us to assess biological and experimental variability. For a subset of proteins, enrichment factors in membrane fractions (E_M _and E_IM _values, Additional Files 1 and 2) were calculated. The combination of this data served to establish membrane protein categories and was compared to the prediction of bioinformatic motifs indicative of membrane association of proteins (Additional Files 1 and 2). Membrane proteins with conserved motifs in Gram-negative bacteria comprise integral IM proteins with α-helical TMDs, integral OM proteins with *β*-barrel structures and lipid-anchored peripheral membrane proteins. Generally conserved structural motifs are not found among other peripheral membrane proteins. Interestingly, proteins thought to bind non-covalently to the periphery of membranes showed considerable variation in their quantitative distribution among membrane fractions which resulted in their assignments to three different membrane protein groups (i-M, p-M and p-M* categories, Additional File 2). More than 300 membrane-associated proteins listed in that Table are displayed in the Additional Files 3, 4, 5, 6, 7, 8, 9 and 10 with equivalent spot numbers.

### Peripheral membrane proteins with moderate to high solubility in aqueous solutions

**Figure 3:**
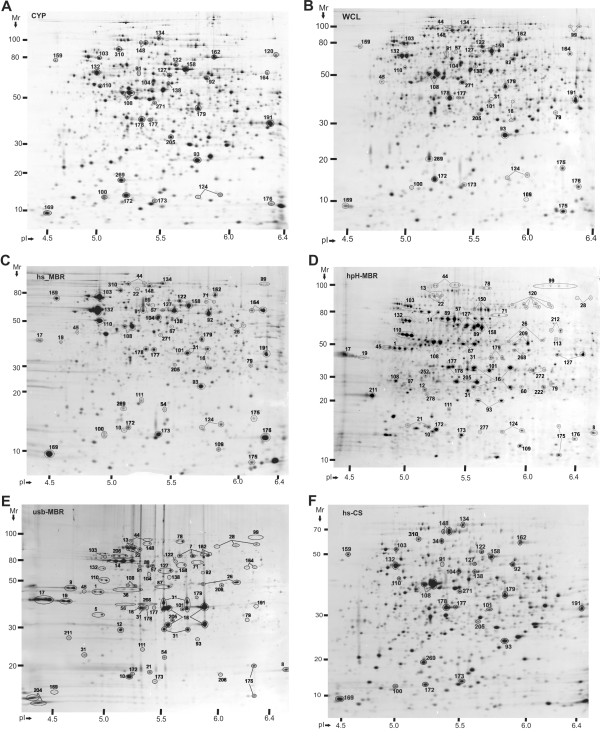
**A comparison of spot profiles in 2D gels derived from a *Y. pestis *KIM6+ whole cell lysate and five subcellular fractions**. Acronyms are described in the flowchart of Figure 1. Cells were grown to stationary phase at 26°C. First dimension IEF separations were performed in the pH range from 4 to 7. The M_r _range of second dimension SDS-PAGE separations was 10–200 kDa. Gel image analysis details are provided in the text. Spot identifications by MS confirmed appropriate spot matching. Spot numbers are equivalent to those denoted in Table 1; Additional File 2.

Proteins enriched in hs-MBR and hpH-MBR fractions, but also detected in soluble CYP or PPS fractions, comprised 99 entries in Additional File 2 (p-M category). In quantitative terms, E_M _and E_IM _values fell into the ranges of 0.05 to 1.2 and 0.3 to 4, respectively. Their quantitative distribution among membrane fractions is compatible with the general definition of peripheral membrane proteins. These proteins appeared to associate with membranes temporarily, did not require detergents for solubilization and were also present in cytoplasmic and/or periplasmic fractions. High salt and high pH membrane extraction conditions favor the solubilization of proteins bound to membranes electrostatically. Judged from their abundance in CYP *vs*. PPS fractions and the presence of export signal sequences, most of these proteins should localize at the cytoplasmic surface of the IM. Many of these proteins are also predicted to be peripheral membrane components of multi-subunit complexes and associate with other integral membrane proteins. Examples are NuoC (#71), LpdA (#92), ManX (#101), AdhE (#99), AtpD (#110), AtpA (#127) and GuaB (#164). All protein numbers referenced here and in the following two sections pertain to Figure 3, unless otherwise stated. Such protein complexes, often characterized in *E. coli*, include ATP synthase (AtpA and AtpD), NADH dehydrogenase (NuoC), pyruvate dehydrogenase (LpdA) and a mannose-specific phosphotransferase system (ManX).

Orthologs of proteins known to associate peripherally with membranes in *E. coli *are the phage shock protein PspA, the sigma factor RpoE and the cell division protein FtsZ (#45). A characterized peripherally OM/cell surface-associated protein of *Y. pestis *is the Caf1 antigen. This protein is expressed only at 37°C and was highly enriched in the hs-CS fraction of *Y. pestis *cells grown at 37°C (#29; Additional File 3). The protease DegQ and the catalase KatY were enriched in hs-MBR and hpH-MBR fractions (#113 and #120, respectively), and even more abundant in the hs-MBR fraction of *Y. pestis *cells grown at 37°C (Additional File 4). Both proteins are involved in responses to cellular stress, supporting the notion that peripheral membrane association of proteins is influenced by environmental factors such as temperature. Both proteins have export signal sequences and were profiled in PPS fractions, suggesting attachment to the IM or OM in the periplasm. An uncharacterized protein particularly enriched in the hpH-MBR fraction was the probable N-acetylmuramoyl-L-alanine amidase y1845 (#205).

The assignment of 28 proteins to the membrane periphery of the IM or OM was more tentative (p-M* category, Additional File 2). While consistently observed in at least two membrane fractions, similar to proteins assigned to the p-M category, these proteins were more abundant in CYP or PPS fractions. In quantitative terms, E_M _and E_IM _values fell into the ranges of 0.01 to 0.07 and 0.3 to 4, respectively. Examples are AhpC (#93), TufB (#108), GroEL (#132) and the virulence factor Ymt (#158). Some of these proteins may associate with membranes in the form of phospholipid-protein microdomains, possibly under thermal or oxidative stress conditions (*e.g*. AhpC, GroEL, DnaK, HtrA and Lon). The elongation factor Tu (TufB) reportedly has two functions in rod-shaped bacteria, as an enzyme in ribosomal protein biosynthesis and as a protofilament-forming protein of the cytoskeleton. The cytoskeleton is physically associated with the cytoplasmic surface of the IM, a characteristic that could explain why TufB was consistently detected in all three membrane fractions. In support of this, three other cytoskeleton-associated proteins (MreB, MinD and FtsZ) were enriched in membrane fractions. Ymt, a phospholipase D, was reported to be present intra- and extracellularly in the *Y. pestis *flea environment [14]. Our data indicate that Ymt was released in moderate quantities from cells at 26°C *in vitro *(#158, hs-CS fraction) and present in all other subcellular fractions. This virulence factor may transiently reside in membranes during a protein export process yet to be investigated. In summary, proteins in the p-M* category are compatible with the general definition of peripheral membrane proteins, but relatively low enrichment levels in membrane fractions resulted in lower confidence for this subcellular protein designation. Most proteins assigned to this category are not known to participate in multi-subunit membrane protein complexes.

### Cytoplasmic and ribosomal membrane-contaminating proteins

Eighty proteins were frequently profiled in all three membrane fractions, but in much lower quantitative ratios compared to the CYP fraction (E_M _values <0.04) and the PPS fraction, and not with the same level of reproducibility as proteins sorted into the p-M and p-M* categories. Examples are TktA (#162), CysK (#178), Fba (#179), GapA (#191) and Tpx (#269). Although clear evidence is missing, we assume that protein denaturation and aggregation in cell lysates caused unspecific binding of these proteins to membranes and co-fractionation of protein aggregates with membranes. The fact that this group of proteins includes numerous highly abundant cytoplasmic proteins, *e.g*. those involved in carbon metabolism, supports a positive relationship between protein abundance and membrane contamination levels. Several ribosomal proteins (*e.g*., RplL, #169 and RplI, #176, RpsA, #103 and RpsF, #173) featured E_M _values similar to those of true peripheral membrane proteins. While strictly experimental criteria place these proteins in the p-M* category, they were designated here as membrane contaminants. Ribosomes are organelle-like structures that may co-fractionate with membranes via centrifugation following *Y. pestis *cell lysis. A few ribosomal and soluble cytoplasmic contaminant proteins are included in Additional File 2, more of them in Additional File 1; the protein category is termed C/R.

### Integral membrane proteins and peripheral membrane proteins with low solubility in aqueous solutions

Proteins enriched in hpH-MBR and usb-MBR fractions were initially designated integral membrane proteins. E_M _values were often not available because these proteins were not detected in CYP fractions. E_IM _values were calcuated occasionally, ranging from 4 to 70. These proteins comprise 220 entries in Additional File 2 (categories i-M and i-OM). Search results with the algorithms TMHMM and BOMP supported experimental assignments of integral membrane proteins in many, but not in all cases. Lipid-anchored proteins, which have a conserved motif surrounding a cysteine residue that becomes the lipid anchor following signal peptide cleavage, were also strongly enriched in hpH-MBR and usb-MBR fractions. While such lipoproteins favor attachment to the IM and/or OM via fatty acylation in the periplasmic space, their topographies resembles those of other peripheral membrane proteins. Examples are Lpp (Braun's lipoprotein in *E. coli*), Pal and AcrA (#204, #21 and #36, respectively; Figure 3E). Lpp, not recognized as an ORF in the *Y. pestis *KIM genome, and Pal tether the peptidoglycan to the OM of Gram-negative bacteria. *E. coli *AcrA is a protein anchored in the IM and part of the multidrug efflux system AcrAB-TolC. Nearly 40 proteins with conserved glycerolipid acylation sites were identified and assigned to the i-M or i-OM categories. Spot resolution of these lipoproteins in 2D gels was nearly as poor as that of proteins with two or more TMDs.

Surprisingly, 42% of the proteins listed in the i-M category lacked a predicted IM or OM integration motif, even though they were highly enriched in hpH-MBR and usb-MBR fractions. Of note, a highly conserved protein family structurally characterized in Gram-negative bacteria was well represented among these proteins: ATP-binding subunits of ABC transporters. These proteins are often described as dimeric peripheral IM subunits of ABC transporters that also harbor two cognate, integral membrane permease subunits. The solubilization traits of ATP-binding subunits of ABC transporters are not in good agreement with the general definition of peripheral membrane proteins. They do not appear to be very water-soluble, considering their absence in cytoplasmic fractions and low extraction levels in hs-MBR fractions. Their enrichment in hpH-MBR and usb-MBR fractions suggests permanent membrane attachment. Abundant ATP-binding subunits were ProV (#67), involved in amino acid import, MalK (#209), involved in sugar import and YfeB (#272), involved in iron/manganese import. Twenty-five additional (putative) ATP-binding subunits were identified, totaling 28% of all of these subunits of ABC transporters predicted by the database TransportDB for the *Y. pestis *KIM genome. Many other proteins devoid of conserved motifs indicative of IM or OM integration showed similar enrichment characteristics in membrane-extracted fractions and, in cases where *E. coli *orthologs were characterized, also appeared to participate in membrane protein complexes. Examples are FtsA, MinE and MinD (#252), all of which are peripheral membrane components of the cell division apparatus. Many of the aforementioned proteins tended to be more enriched in hpH-MBR than in usb-MBR fractions, indicative of their extractability from membranes without detergents. In this regard, the proteins met the definition of peripherally membrane-attached proteins. Like *β*-barrel OM proteins and water-soluble peripheral membrane proteins, 2D spots of these proteins were generally well resolved.

Integral membrane proteins featuring TMDs were of low abundance in 2D gels and yielded low sequence coverage by MS. Even proteins part of abundant IM-localized protein complexes (*e.g*. the subunit AtpB of ATP synthase) were not detected as distinct spots, demonstrating the technical difficulties to resolve these hydrophobic proteins in 2D gels. Fragments of TMD proteins profiled in hpH-MBR and usb-MBR fractions were often detected as low-abundance spots. Only one protein with several α-helical TMDs was clearly visualized in 2D gel images, the metal ion-transporting ATPase ZntA (#266). Based on the number of proteins with two or more predicted TMDs (> 660 ORFs in the *Y. pestis *KIM genome), less than 5% (32 entries, Additional File 2) were profiled in 2D gel-based experiments. Our methodology was ineffective to survey integral IM proteins comprehensively. Finally, the detection of tryptic peptides in proximity to the proteins' N-termini was indicative of IM localizations as OM proteins lose their N-terminal signal sequences during export into the cell envelope. Such peptides were observed for forty i-M category proteins (Additional File 1).

Beta-barrel OM proteins resolved well in 2D gels and were partially extracted in hpH-MBR fractions, but were more enriched in usb-MBR fractions. The algorithm BOMP predicted *β*-barrel structures for 38 proteins associated with i-M/i-OM categories (Additional File 1). Orthologs of many characterized *E. coli β*-barrel proteins were identified, supporting the notion that the *Y. pestis *OM proteome survey was comprehensive. This included OmpA (#16), OmpF (#17), OmpC (#19), YaeT (#22) and TolC (#26). OM protein profiling is of interest because several of these proteins have been linked to *Y. pestis *virulence in humans, *e.g*. the TonB-dependent receptor for yersiniabactin and pesticin (#14), the protease Pla (#31) and the adhesion protein Ail (#8). The putative porin y2983 deserves re-annotation in the *Y. pestis *database as a phosphate- or anion-specific OM porin. It was expressed only in P_i_-starved cells at 26°C and strongly enriched in the usb-MBR fraction (spot #20; Additional Files 6 and 7). A functional role in P_i _import was supported by the presence of a conserved PHO box upstream of the y2983 gene (position -121 to -104; TTGTCATAAATATATCAC). The PHO box is the binding site of PhoB/R, a two-component regulator for P_i _acquisition in *E. coli*.

**Figure 4:**
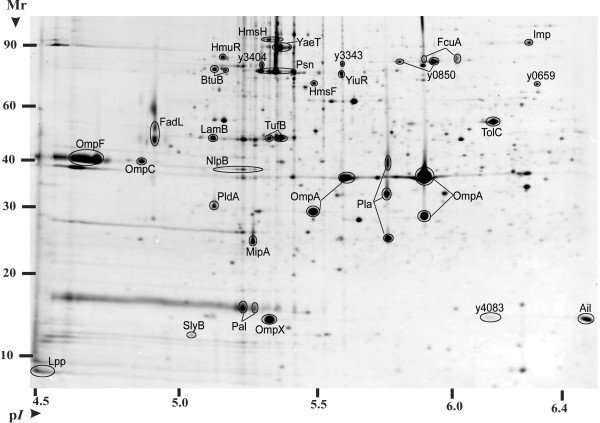
**Protein display for an OM-enriched sucrose density gradient fraction derived from stationary phase *Y. pestis *cells grown at 26°C**. A crude membrane fraction was layered on top of a 3-step sucrose density gradient (15%, 54% and 70% w/V sucrose) and centrifuged at 105,000 × g for 17 h at 5°C. A whitish OM-enriched band was isolated, washed with a 2.5 M NaBr solution and extracted with 8 M urea/2 M thiourea/1% ASB-14 (usb-MBR fraction). *Circa *120 *μ*g of the protein extract was analyzed in a 2D gel as described in the legend of Figure 3. OM proteins are denoted with their short names or gene locus tags.

Specific localization assignments were made for OM proteins (i-OM category). In an experiment repeated three times, OM fractions were isolated via sucrose density gradient centrifugation and extracted. Protein spots more abundant in OM-enriched usb-MBR fractions compared to mixed membrane usb-MBR fractions were identified by MS. Such protein spots collapsed into 31 distinct gene products and amounted to *ca*. 90% of the volumetric intensity of all spots in the respective 2D gel (n = 3). They were designated integral OM proteins and are displayed and annotated in Figure 4. Six structural types of *β*-barrel OM proteins were represented: 1. small, monomeric and peptidoglycan-linked (*e.g*. OmpA); 2. small, monomeric and cell surface-associated (*e.g*. Ail, OmpX); 3. dimeric and with enzymatic activity (*e.g*. PldA); 4. trimeric, general diffusion porins (*e.g*. OmpC, OmpF); 5. trimeric, substrate-specific porins (*e.g*. FadL, LamB); 6. TonB-dependent receptors (*e.g*. Psn, HmuR).

### High M_r _membrane protein complexes

**Figure 5:**
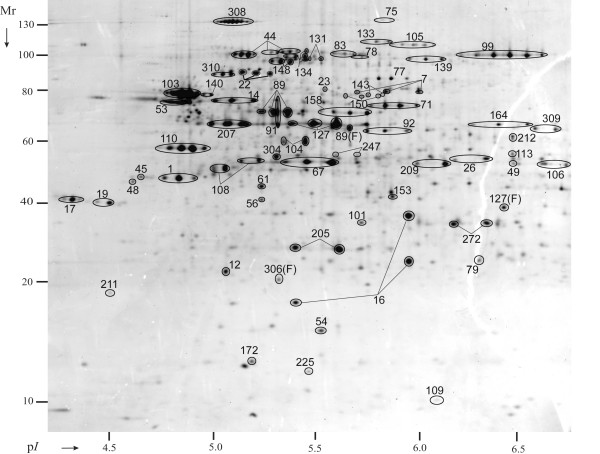
**Protein spot display for a high M_r _fraction derived from alkaline membrane extraction of *Y. pestis *KIM6+ cells grown to stationary phase at 26°C**. Proteins isolated from the hpH-MBR fraction were loaded onto a Superdex 200 column (1.6 × 100 cm), separated in the presence of 0.05% Triton X-100, concentrated to *ca*. 1 mg/mL and lyophilized for 2D gel analysis. The fraction displayed here corresponded to a native protein M_r _range from *ca*. 250 to 450 kDa. Spot numbers are equivalent to those denoted in Additional File 2.

To address the concern that cell lysis followed by protein aggregation severely interfered with reliable assignments of membrane-associated proteins, particularly those localized in the membrane periphery, our intention was to show that partially intact membrane protein complexes could be isolated from membrane fractions of cell lysates in high M_r _ranges. This would be indicative of native protein states. The hpH-MBR fraction derived from *Y. pestis *KIM6+ cells grown at 26°C was isolated in two experimental replicates. Proteins were maintained in a soluble state with 0.075% Triton X-100 and SEC-fractionated. Five fractions each representing a different M_r _range (500 to 20 kDa) revealed different protein patterns in 2D gels. High M_r _fractions in the range above 250 kDa were analyzed by MS. *Circa *50 orthologs of proteins known to participate in multi-subunit membrane complexes in *E. coli *were indeed profiled in the high M_r _range. Proteins are listed in the 'MPC' column of Additional File 2. Proteins are displayed in Figure 5 with equivalent spot numbers. YaeT (#22) and NlpB (#56) are part of the OM protein assembly complex. LamB (#1) is the trimeric OM maltoporin. IM protein complexes implicated in energy metabolism and conservation are the F_1_-ATP synthase sub-complex α_3_, *β*_3_, δ, γ, ε (α, *β *and ε subunits; spots #127, #110 and #109, respectively), pyruvate dehydrogenase (AceE, AceF and LpdA; spots #134, #91 and #92, respectively), NADH dehydrogenase (NuoC and NuoG; spots #71 and #83, respectively) and the dimeric alcohol dehydrogenase AdhE (#99). ATP-binding subunits of ABC transporters (ProV, #67; MalK, #209; YfeB, #272) and proteins linked to the cytoskeletal network and cell division (FtsA, #247; FtsZ, #45; MreB, #61; EF-Tu, #108) were also identified. Among the proteins not characterized to date as components of membrane-associated complexes in *Y. pestis *were putative type VI secretion system subunits (ClpB, #78, y3673, #172; y3674, #89; y3675, #211) and a putative phospholipid-binding protein (y2104, #207). While we only infer the association of aforementioned proteins with distinct membrane protein complexes, this experiment was in support of the notion that many proteins assigned to the membrane periphery were indeed enriched in high M_r _fractions, while proteins designated membrane contaminants were not.

### Proteins with several transmembrane domains identified via 2D-LC-MS/MS

In order to profile more *Y. pestis *proteins featuring α-helical TMDs, we resorted to a 2D-LC-MS/MS approach (experiment performed in triplicate). Following extensive wash and pre-digestion steps to reduce the quantities of soluble contaminant and peripheral membrane proteins, *Y. pestis *membrane fractions derived from cells cultured at 26°C were subjected to treatment with trypsin/chymotrypsin, and the resulting peptide mixtures were analyzed. *Circa *55% of the proteins designated as membrane-associated via 2D gel profiling were also identified by 2D-LC-MS/MS. Analysis with the TMHMM algorithm revealed that, with an overlap of 22 proteins, 32 and 116 proteins with more than one TMD were identified via the 2D gel and 2D-LC-MS/MS methodologies, respectively (Venn diagram, Figure 2B). The latter methodology clearly improved our ability to detect integral IM proteins. Membrane transporters were the dominant functional protein group found in the 2D-LC-MS/MS dataset. According to the TransportDB database, 25 transporter families residing in the IM of *Y. pestis *KIM were represented. This included 14 ABC transporter permeases, a variety of ion channels, two sugar-specific IM subunits of phosphotransferase systems and various secondary transporters, such as major facilitator superfamily (MSF) and amino acid-polyamine organocation (APC) transporters. Proteins only identified by 2D-LC-MS/MS and showing evidence for membrane association based on PSORTb and TMHMM searches are listed in part II of Additional File 2. Numerous ribosomal proteins were also retained in the membrane fraction and profiled by 2D-LC-MS/MS raising further questions about natural membrane association *vs*. unnatural co-aggregation of ribosomes with membranes in cell lysates.

## Discussion

The cell envelope of Gram-negative bacteria is composed of the IM, the periplasmic space, the peptidoglycan meshwork and the OM [48]. In an extension of a recent study of the periplasmic proteome of a derivative of the *Y. pestis *KIM strain [37], the membrane proteome of this strain was profiled here. We used a differential 2D gel display approach comparing periplasmic, cytoplasmic and sequentially extracted membrane fractions to discern various types of membrane-associated proteins, primarily using *Y. pestis *cells grown to stationary phase at 26°C, the ambient temperature of the flea vector. In order to increase the number of proteins with hydrophobic TMDs, a 2D-LC-MS/MS analysis was performed using fractions specifically enriched in integral membrane proteins.

### Peripheral membrane proteins

Peripheral membrane proteins are generally defined as water-soluble proteins that bind temporarily to membrane surfaces, often to other integral membrane proteins and often as subunits of membrane protein complexes. Exceptions are lipoproteins, proteins that are permanently attached to membranes via glycerolipid acylation. Water-soluble peripheral membrane proteins can be extracted from membrane pellets with high salt solutions or at a high pH. Using such extraction conditions, we identified nearly 130 proteins, designated here as peripheral membrane proteins with moderate to high solubility in aqueous solutions. These proteins were discriminated from membrane-contaminating cytoplasmic and periplasmic proteins based on their relative enrichment in membrane *vs*. soluble fractions. Membrane contamination with soluble proteins is unavoidable during the subcellular fractionation of cell lysates, a topic previously referred to in the results section. Information on characterized protein orthologs, particularly from *E. coli*, strongly supported the assignments of water-soluble peripheral *Y. pestis *membrane proteins. Among the highly abundant proteins were subunits of several membrane protein complexes linked to energy metabolism, *e.g*. ATP synthase, NADH dehydrogenase, pyruvate dehydrogenase, 2-oxoglutarate dehydrogenase, succinate dehydrogenase and fumarate reductase. Response regulators of two-component regulatory systems, *e.g*. PhoB, PhoP and CpxR, were also assigned to this group of proteins. They seem to bind temporarily to the cytoplasmic side of the IM where they interact with cognate integral membrane sensory proteins. Two recent studies focused on the identification of intact *E. coli *membrane protein complexes. In one study, protein complexes were dissociated with small alcohols followed by diagonal electrophoresis [49]. *Circa *50% of the identified *E. coli *proteins were orthologs to proteins associated with the *Y. pestis *membrane periphery in our survey. In the second study, 2D blue native gels were used to isolate membrane protein complexes [50], and 58% of the identified *E. coli *proteins were orthologs to proteins we assigned to the *Y. pestis *membrane periphery. Finally, more than 50 of the *Y. pestis *proteins assigned to the membrane periphery, derived from high pH membrane extracts, fractionated into high M_r _fractions (> 250 kDa) via SEC. This data supported the notion that many peripheral membrane proteins can be recovered from cell lysates as components of intact or partially intact membrane protein complexes.

In addition to *ca*. 40 lipoproteins, more than 90 proteins devoid of conserved membrane integration motifs were extracted at a high pH and in membrane-denaturing solutions. These proteins were not or barely detected in high salt-extracted membrane and cytoplasmic fractions, and were designated peripheral *Y. pestis *membrane proteins with low solubility in aqueous solutions. Several orthologs of such proteins have been described as peripheral membrane proteins in the literature, *e.g*. various ATP-binding subunits of ABC transporters and the cell division proteins MinD, MinE and FtsA. There is evidence that some of these proteins specifically bind to membrane phospholipids, *e.g*. MinD [51]. We speculate that, unlike typical peripheral membrane proteins that bind to integral membrane proteins via electrostatic interactions and are water-soluble, proteins with such phospholipid-binding motifs are more difficult to extract from membanes and reside only in small quantities in soluble form, for instance, in the cytoplasm. The structure of monotopic integral membrane proteins would also explain the low solubility in high salt membrane extracts, but the existence of monotopic membrane proteins in bacteria has not been confirmed. The structures and topographies of several bacterial ATP-binding subunits of ABC transporters have been solved, and there is little evidence for direct interactions between them and membrane phospholipids [52]. The examination of phospholipid-binding motifs in less water-soluble peripheral membrane proteins is an interesting research topic.

The concept of temporary membrane association of stress response and cytoskeletal network-associated proteins is a research topic also deserving more attention. Heat shock and oxidative stress response proteins were associated in this survey with the *Y. pestis *membrane periphery. These proteins were generally quite abundant as soluble proteins (*e.g*. DnaK, HtpG, ClpB, Lon, KatY, DegQ, UspA and Dps). *E. coli *orthologs of some of these proteins seem to be involved in protein aggregate repair and solubilization [47] and in the restoration of membrane functionality during cellular stress such as elevated temperatures [53]. Indeed, several of the *Y. pestis *stress response proteins were more abundant in membrane fractions of cells grown at 37°C than at 26°C. Evidence for the interaction of heat shock proteins with membrane lipid microdomains, also called lipid rafts, is emerging. Such interactions are hypothesized to facilitate extracellular release and immuno-stimulatory activities of the proteins [53,54]. Stress response proteins have indeed been identified as major antigens in various organisms. For a cytoplasmic protein, the elongation factor Tu (TufB) was unusually abundant in all membrane fractions. Its abundance may be relevant in the context of data describing *E. coli *TufB as an intracellular protofilament protein, a key component of the cytoskeleton [55]. Orthologs of other less abundant membrane-associated *Y. pestis *proteins identified in this survey also participate in the assembly of the *E. coli *cytoskeletal network, *e.g*. bacterial tubulin (FtsZ), bacterial actin (MreB) and the ATPase MinD [56].

### Integral membrane proteins and protein complexes of the IM

Use of the 2D-LC-MS/MS strategy resulted in nearly four times as many identifications of proteins with two or more α-helical TMDs than proteomic analysis in 2D gels, accounting for *ca*. 20% of the bioinformatically predicted TMD protein subset in the *Y. pestis *KIM genome. The majority of the proteins were involved in transport functions. The largest conserved family of IM-localized transporters are ABC transporters. Some 30% of their *in silico*-predicted ATP-binding subunits, peripheral membrane proteins, and 10% of their *in silico*-predicted permease subunits, integral membrane proteins, were identified here. Among these proteins were subunits of three characterized *Y. pestis *ABC transporters (Yiu, Yfe, Yfu), all of which are implicated in iron acquisition [19,57]. Cognate solute-binding proteins for all of the identified ABC transporters were profiled in a recent study of the *Y. pestis *periplasmic proteome where identical cell growth conditions were applied [37]. Expression of ABC transporters is typically induced when cells sense nutrient starvation. Transcriptional regulation mechanisms pertaining to ABC transporters are quite complex. It was shown here that *Y. pestis *PstB, the ATP-binding subunit of a high affinity phosphate ABC transporter, was expressed only in P_i_-starved cells. The most abundant ABC transporters in stationary phase *Y. pestis *cells were MalK and ProV, indicative of intracellular maltose and proline shortages, respectively. Both proteins were detected in high M_r _fractions of membrane extracts suggesting at least partially intact ABC transporter complexes. The two orthologous *E. coli *ABC transporters (MalFGK_2 _and ProV_2_W_2_) were recently isolated as intact tetrameric membrane-associated complexes [50]. The variety of IM-associated ion channels and small molecule transporters surveyed in the *Y. pestis *KIM membrane proteome was remarkable (25 protein families). A smaller subset of IM-associated transporters and ion channels was recently profiled in the *Y. pestis *KIM5 strain [24]. Finally, several uncharacterized *Y. pestis *proteins were identified in high M_r _membrane fractions and appeared to be components of multi-subunit membrane protein complexes. Examples are the proteins y3674, y3675 and ClpB2, all part of a putative type VI secretion system which has been linked to pathogenicity of *Vibrio cholerae *[58], and the protein y2104, a putative phospholipid-binding protein whose ortholog YdgA also formed oligomeric structures in *E. coli *[50].

### OM-associated proteins and protein complexes of the OM

Thirty-one proteins were designated *Y. pestis *OM proteins following the isolation and proteomic analysis of OM-enriched sucrose gradient fractions. A high dynamic range of protein abundances was observed. Bioinformatic predictions for *β*-barrel and lipoprotein motifs suggested OM localizations for *ca*. 20 additional proteins, making this effort the most extensive OM proteome analysis reported to date for a Gram-negative bacterium. All five proteins orthologous to components of the *E. coli β*-barrel OM protein assembly apparatus [59] were identified here; the lipoproteins y1356 (YfgL), y0911 (YfiO), NlpB and SmpA and the *β*-barrel protein YaeT. Several OM proteins have functional roles in microbial pathogenesis and are of particular interest as drug and vaccine targets. Relatively few *Y. pestis *OM proteins have been subjected to characterization efforts, among them are Pla, Ail, Pal, Lpp and SlyB [60,61]. Ail is a small *β*-barrel protein involved in adhesion and serum resistance using *in vitro *assays [62,63]. Ail was shown here to be a highy abundant OM protein in cells grown at 37°C. Additional small *β*-barrel proteins whose functional roles are unknown in the flea vector and mammalian hosts were identified; OmpX, MipA and the putative phospholipase PldA. The TonB-dependent OM receptor Psn was highly abundant in OM surveys at 26 and 37°C and has been implicated in iron/siderophore import and virulence in mammalian hosts. Other TonB-dependent OM receptors, nine of which were identified in *Y. pestis *membrane fractions, have not been functionally characterized to date.

## Conclusion

A comprehensive membrane proteome analysis of the Gram-negative bacterium Y. pestis is presented. More than 450 Y. pestis proteins were sequentially extracted from membrane fractions, identified by MS and divided into different membrane protein association categories. Thirty-one proteins were specifically associated with the OM and represent interesting targets with respect to potential roles in the virulence of Y. pestis in the flea and/or the mammalian hosts. Numerous peripheral membrane proteins appeared to be associated with high Mr protein complexes. This data yields valuable information for the improvement of membrane protein annotations in Y. pestis genome databases.

## Competing interests

The authors declare that they have no competing interests.

## Authors' contributions

RP: primary role in designing the study, analyzing and interpreting the data and writing the article; STH: quantitative analysis of the data, bioinformatic analyses, database queries, design of figures for the article; DJC: acquisition of the majority of the LC-MS/MS data; JMR: acquisition of 2D gel data and isolation of high M_r _membrane protein fractions; HA: acquisition of the majority of the MALDI-MS; PPP: responsible for the development of the 2D gel methodology, acquisition of 2D data and interpretation of the results; MS: performed an extensive analysis of the *Y. pestis *membrane fraction by 2D-LC-MS/MS; SK: assistance in the acquisition of MALDI-MS and LC-MS/MS data and in the interpretation of the biological significance of the data; MJS: performed the 2D-LC-MS/MS analysis; CLB: responsible for the development of the MS methodologies in the early phase of the project; RDP: participated in the design of the study and the interpretation of biological significance the data, provided strains, methods and advice for cell growth conditions; RDF: participated in the design of the study; SNP: participated in the design and review of the study.

## Supplementary Material

Additional file 1 **Supplemental Table**. Supplemental table with extensive information on *Yersinia pestis *KIM proteins identified from membrane fractions: protein annotations, functional role categories, differential 2D gel display and mass spectrometry data. Click here for file

Additional file 2 **Table 1**. Table lists all identified *Yersinia pestis *KIM6+ membrane-associated proteins with 2D gel spot numbers, locus tags, gene names, their detection in high M_r _membrane fractions, their categorization, functional role descriptions, and enrichment factors in membrane fractions (E_M _and E_IM _values). Click here for file

Additional file 3 **Supplemental Information (Figure Two)**. Protein display of the high salt-extracted membrane fraction isolated from *Y. pestis *KIM6+ cells grown at 26°C (pH range of 4–6.5). Click here for file

Additional file 4 **Supplemental Information (Figure Three)**. Protein display of the high salt-extracted membrane fraction isolated from *Y. pestis *KIM6+ cells grown at 37°C (pH range of 4–9). Click here for file

Additional file 5 **Supplemental Information (Figure Four)**. Protein display of the high pH-extracted membrane fraction isolated from *Y. pestis *KIM6+ cells grown at 26°C (pH range of 4–6.5). Click here for file

Additional file 6 **Supplemental Information (Figure Five)**. Protein display of urea/sulfobetaine-14-extracted membrane fraction isolated from *Y. pestis *KIM6+ cells grown in low phosphate media at 26°C (pH range of 4–6.5). Click here for file

Additional file 7 **Supplemental Information (Figure Six)**. Protein display of urea/sulfobetaine-14-extracted membrane fraction isolated from *Y. pestis *KIM6+ cells grown in low phosphate media at 37°C (pH range of 4–6.5). Click here for file

Additional file 8 **Supplemental Information (Figure Seven)**. Protein display of urea/amidosulfobetaine-14-extracted membrane fraction isolated from *Y. pestis *KIM6+ cells grown to stationary phase at 26°C (pH range of 4–7). Click here for file

Additional file 9 **Supplemental Information (Figure Eight)**. Protein display of urea/amidosulfobetaine-14-extracted membrane fraction isolated from *Y. pestis *KIM6+ cells grown at 37°C (pH range of 6–10). Click here for file

Additional file 10 **Supplemental Information (Figure One)**. Protein display of the high salt-extracted cell surface fraction isolated from *Y. pestis *KIM6+ cells grown at 37°C (pH range 3.5–10). Click here for file
