# Supplementary material for: Integral and peripheral association of proteins and protein complexes with Yersinia pestis inner and outer membranes
Source: Proteome Sci. 2009 Feb 19;7:5. doi: 10.1186/1477-5956-7-5 (PMC2663777; doi:10.1186/1477-5956-7-5)
Supplement: Additional file 2 — Table 1. Table lists all identified Yersinia pestis KIM6+ membrane-associated proteins with 2D gel spot numbers, locus tags, gene names, their detection in high Mr membrane fractions, their categorization, functional role descriptions, and enrichment factors in membrane fractions (EM and EIM values). [file 1477-5956-7-5-S2.doc]

## Table 1. The *Yersinia pestis* KIM6+ membrane proteome: protein annotations and descriptions, their presence in high M_r_ membrane fractions, protein categorization and enrichment in membrane fractions.

| **Part I. Proteins analyzed in 2D gels** | | | | | | | | | | | | | | | | | | |  |
| --- | --- | --- | --- | --- | --- | --- | --- | --- | --- | --- | --- | --- | --- | --- | --- | --- | --- | --- | --- |
| a) | b) | | c) | | d) | e) | | f) | g) | | h) | i) | | i) | j) | k) | l) | m) | |
| **Spot No.** | **Locus tag** | | **Gene name** | | **MPC** | **Category** | | **Protein name and description** | **MS score** | | **MS score** | **pI** | | **Mr** | **P-SORTb** | **TMHMM** | **EM** | **EIM** | |
| 209 | y0031 | | malK | | Y | i-M | | maltose/maltodextrin ABC transporter ATP-binding protein | 512 | | 50 | 5.97 | | 40985 | IM | 0 | + |  | |
| 300 | y0036 | | - | |  | i-M | | hemolysin-coregulated protein (HCP) | 142 | |  | 5.46 | | 19202 | U | 0 | + |  | |
| 274 | y0038 | | - | |  | i-M | | hypothetical protein y0038 (putative type VI secretion system subunit) | 232 | |  | 5.46 | | 56211 | U | 0 |  |  | |
|  | y0108 | | (yicH) | |  | i-M | | hypothetical protein y0108 (putative LPS biogenesis protein) | 112 | | 69 | 5.81 | | 62714 | OM | 1 |  |  | |
| 47 | y0118 | | (lppC) | |  | i-M | | glycosylase | 415 | |  | 5 | | 74211 | U | 0 |  |  | |
| 46 | y0123 | | (yraP) | |  | i-M | | hypothetical protein y0123 (putative phospholipid-binding lipoprotein) | 462 | | 1442 | 9.46 | | 21966 | P | 1 | + |  | |
| 225 | y0136 | | (yhcB) | | Y | i-M | | cytochrome d ubiquinol oxidase subunit III | 402 | | 190 | 5.64 | | 15476 | U | 1 | + |  | |
| 284 | y0142 | | (yrbB) | |  | i-M | | hypothetical protein y0142 | 60 | |  | 4.85 | | 11343 | U | 0 | + |  | |
| 226 | y0146 | | (yrbF) | |  | i-M | | putative ABC transporter ATP-binding protein YrbF | 194 | |  | 5.3 | | 29949 | MSL | 0 | + |  | |
| 60 | y0153 | | lptB | |  | i-M | | putative ABC transporter ATP-binding protein YhbG (LptB) | 259 | |  | 6.02 | | 26967 | C | 0 | + |  | |
| 2 | y0184 | | tcbA | |  | i-M | | toxin complex protein | 972 | |  | 5.54 | | 137158 | OM | 0 |  |  | |
| 3 | y0185 | | tcaC1 | |  | i-M | | toxin complex protein | 130 | |  | 5.17 | | 171442 | OM | 0 |  |  | |
| 61 | y0202 | | mreB | | Y | i-M | | regulator of ftsI and penicillin-binding protein 3 | 366 | | 221 | 5.18 | | 37049 | C | 0 | + |  | |
| 238 | y0209 | | accC | |  | i-M | | acetyl-CoA carboxylase biotin carboxylase subunit | 285 | | 143 | 6.38 | | 49962 | C | 0 | + |  | |
|  | y0329 | | (ytfR) | |  | i-M | | ATP-binding component of ATP transport system | 117 | |  | 5.93 | | 54564 | MSL | 0 |  |  | |
|  | y0363 | | wzzE | |  | i-M | | transport protein (lipopolysaccharide biosynthesis) | 173 | | 773 | 5.72 | | 39850 | IM | 2 |  |  | |
|  | y0378 | | hemY | |  | i-M | | putative protoheme IX biogenesis protein | 143 | | 73 | 8.71 | | 45951 | C | 2 |  |  | |
| 48 | y0379 | | hemX | | Y | i-M | | uroporphyrinogen III methylase | 511 | | 190 | 4.76 | | 41948 | U | 1 |  | 26.6614 | |
|  | y0393 | | corA | |  | i-M | | magnesium and cobalt permease (cation transport) | 81 | |  | 4.72 | | 38488 | C | 2 |  |  | |
|  | y0407 | | - | |  | i-M | | hypothetical protein y0407 (putative inner membrane lipoprotein) | 226 | | 134 | 5.8 | | 18869 | U | 0 |  |  | |
| 266 | y0410 | | zntA | |  | i-M | | zinc, lead, cadmium, and mercury transporting ATPase | 84 | |  | 5.7 | | 84427 | IM | 6 |  |  | |
| 270 | y0416 | | ftsY | |  | i-M | | cell division membrane protein | 441 | |  | 4.45 | | 60150 | IM | 0 |  |  | |
|  | y0418 | | ftsX | |  | i-M | | cell division protein FtsX | 62 | |  | 7.82 | | 34883 | IM | 4 |  |  | |
| 131 | y0442 | | metE | | Y | i-M | | 5-methyltetrahydropteroyltriglutamate-homocysteine methyltransferase | 395 | | 235 | 5.44 | | 86555 | C | 0 | + |  | |
| 35 | y0448 | | (rumC) | |  | i-M | | alpha helix chain (putative DNA recombination protein) | 681 | |  | 5.81 | | 57455 | IM | 1 |  |  | |
|  | y0539 | | hmuV | |  | i-M | | ATP-binding protein of ABC transporter | 105 | |  | 8.31 | | 25806 | U | 0 |  |  | |
| 62 | y0555 | | terZ | |  | i-M | | putative tellurium resistance protein | 452 | | 118 | 6.1 | | 23001 | C | 0 | + |  | |
| 227 | y0632 | | hflX | |  | i-M | | putative GTPase HflX (protease subunit of lambda cII repressor) | 401 | |  | 6 | | 48172 | C | 0 |  |  | |
| 49 | y0633 | | hflK | | Y | i-M | | FtsH protease regulator HflK | 377 | | 415 | 7.79 | | 45258 | U | 1 |  |  | |
| 50 | y0634 | | hflC | |  | i-M | | FtsH protease regulator HflC | 393 | | 560 | 7.85 | | 37414 | U | 1 |  |  | |
| 51 | y0661 | | (ytfN) | |  | i-M | | hypothetical protein y0661 | 505 | | 136 | 7.14 | | 140006 | U | 1 |  |  | |
| 153 | y0664 | | fbp | | Y | i-M | | fructose-1,6-bisphosphatase | 590 | | 321 | 6.03 | | 41455 | U | 2 | + |  | |
| 119 | y0678 | | dacB | |  | i-M | | D-alanyl-D-alanine carboxypeptidase (penicillin-binding protein 4) | 373 | |  | 9.06 | | 52429 | U | 0 |  |  | |
| 228 | y0682 | | hflB | |  | i-M | | ATP-dependent inner membrane metalloprotease | 173 | | 974 | 5.76 | | 71208 | IM | 2 |  |  | |
|  | y0685 | | secG | |  | i-M | | preprotein translocase subunit SecG | 68 | | 181 | 5.05 | | 11623 | IM | 2 |  |  | |
|  | y0695 | | (nlpI) | |  | i-M | | lipoprotein NlpI | 68 | |  | 4.72 | | 33834 | U | 0 |  |  | |
|  | y0702 | | (acrA2) | |  | i-M | | acridine efflux pump (drug transport) | 81 | | 63 | 5.71 | | 41031 | IM | 0 |  |  | |
|  | y0704 | | (ibeB) | |  | i-M | | outer membrane efflux pump protein | 146 | |  | 5.84 | | 49727 | OM | 0 |  |  | |
| 288 | y0735 | | dppD | |  | i-M | | ATP-binding component of dipeptide transport system | 206 | |  | 6.18 | | 33380 | IM | 0 |  |  | |
| 230 | y0795 | | mrcB | |  | i-M | | penicillin-binding protein 1b | 149 | | 111 | 9.43 | | 85641 | MSL | 1 |  |  | |
|  | y0827 | | ftsB | |  | i-M | | cell divison protein FtsB | 205 | | 260 | 5.35 | | 11862 | U | 0 |  |  | |
| 203 | y0833 | | nlpD | |  | i-M | | predicted outer membrane lipoprotein NlpD | 238 | | 112 | 9.52 | | 34917 | U | 0 | + |  | |
|  | y0835 | | mutS | |  | i-M | | DNA mismatch repair protein (MutS-family ATPase) | 216 | |  | 5.69 | | 95039 | U | 0 |  |  | |
| 52 | y0911 | | (yfiO) | |  | i-M | | predicted lipoprotein | 465 | | 108 | 7.71 | | 27542 | U | 0 |  |  | |
| 286 | y0923 | | emrR | |  | i-M | | transcriptional repressor MprA | 114 | | 57 | 8.63 | | 21092 | C | 0 |  |  | |
| 212 | y0951 | | nqrA | | Y | i-M | | Na(+)-translocating NADH-quinone reductase subunit A | 440 | | 62 | 6.42 | | 49989 | U | 0 |  |  | |
|  | y0992 | | yajC | |  | i-M | | preprotein translocase subunit YajC | 104 | | 293 | 9.3 | | 14693 | U | 2 |  |  | |
|  | y0993 | | secD | |  | i-M | | preprotein translocase subunit SecD | 126 | | 582 | 8.8 | | 66861 | IM | 6 |  |  | |
|  | y1021 | | cyoA | |  | i-M | | cytochrome o ubiquinol oxidase subunit II | 130 | | 589 | 8.75 | | 35083 | IM | 3 |  |  | |
|  | y1024 | | (yajG) | |  | i-M | | hypothetical protein y1024 (putative lipoprotein) | 138 | | 290 | 8.66 | | 20866 | U | 0 |  |  | |
| 53 | y1031 | | (ppiD) | | Y | i-M | | peptidyl-prolyl cis-trans isomerase (rotamase D) | 777 | | 541 | 4.95 | | 69849 | U | 1 | + | 4.2284 | |
|  | y1049 | | acrB | |  | i-M | | acridine efflux pump (multidrug transport) | 142 | | 1222 | 5.8 | | 113603 | IM | 11 |  |  | |
| 36 | y1050 | | acrA | | Y | i-M | | acridine efflux pump (multidrug resistance efflux transporter) | 584 | | 595 | 6.1 | | 41921 | IM | 0 | + |  | |
|  | y1170 | | tatE | |  | i-M | | twin arginine translocase protein A | 97 | | 432 | 8.01 | | 8880 | U | 1 |  |  | |
| 198 | y1175 | | dacA | |  | i-M | | D-alanyl-D-alanine carboxypeptidase fraction A | 1445 | | 105 | 8.65 | | 44118 | IM | 0 |  |  | |
| 257 | y1176 | | rlpA | |  | i-M | | rare lipoprotein A | 135 | | 104 | 5.76 | | 38052 | U | 0 |  |  | |
| 258 | y1183 | | rlpB | |  | i-M | | LPS assembly lipoprotein RplB | 278 | | 206 | 6.43 | | 22613 | U | 1 | + |  | |
| 289 | y1186 | | gltL | |  | i-M | | ATP-binding protein of glutamate/aspartate transport system | 77 | |  | 6.23 | | 26914 | C | 0 |  |  | |
|  | y1203 | | nagE | |  | i-M | | N-acetylglucosamine-specific PTS system enzyme IIABC | 119 | | 145 | 5.97 | | 71877 | IM | 9 |  |  | |
| 67 | y1221 | | proV | | Y | i-M | | ATP-binding component of high affinity Gly / betaine / Pro transporter | 301 | |  | 5.88 | | 48729 | C | 0 |  | 20.6764 | |
|  | y1247 | | - | |  | i-M | | voltage-gated potassium channel | 110 | |  | 7.66 | | 43298 | IM | 7 |  | 1.2714 | |
|  | y1296 | | lepB | |  | i-M | | signal peptidase I (leader peptidase) | 108 | |  | 6.9 | | 36811 | IM | 2 |  |  | |
| 81 | y1314 | | - | |  | i-M | | hypothetical protein y1314; putative alpha helix protein | 161 | | 56 | 8.54 | | 39453 | U | 0 |  |  | |
|  | y1321 | | hmpA | |  | i-M | | nitric oxide dioxygenase | 128 | |  | 5.94 | | 45016 | U | 0 |  |  | |
| 231 | y1334 | | (nifS) | |  | i-M | | cysteine desulfurase | 344 | |  | 5.94 | | 45457 | U | 0 |  |  | |
| 264 | y1346 | | yapA | |  | i-M | | autotransporter | 310 | |  | 4.85 | | 149543 | MSL | 1 | + |  | |
|  | y1351 | | (pilF) | |  | i-M | | fimbrial biogenesis protein | 78 | |  | 8.82 | | 27710 | U | 0 |  |  | |
| 141 | y1353 | | ispG | |  | i-M | | 4-hydroxy-3-methylbut-2-en-1-yl diphosphate synthase | 273 | | 74 | 6.56 | | 41058 | C | 0 | + |  | |
| 54 | y1355 | | (yfgM) | | Y | i-M | | hypothetical protein y1355 | 1030 | | 175 | 5.49 | | 22627 | U | 1 | + | 3.5806 | |
| 55 | y1356 | | (yfgL) | |  | i-M | | outer membrane protein assembly complex subunit YfgL | 348 | | 54 | 4.99 | | 42122 | U | 0 | + |  | |
|  | y1376 | | - | |  | i-M | | hypothetical protein y1376 | 184 | |  | 9.54 | | 18811 | U | 0 |  |  | |
| 70 | y1401 | | pstB1 | |  | i-M | | phosphate-specific ABC transporter component | 99 | |  | 6.18 | | 30904 | C | 0 |  |  | |
|  | y1412 | | (yfgC) | |  | i-M | | hypothetical protein y1412 (putative metalloprotease) | 257 | |  | 9.09 | | 57637 | IM | 0 |  |  | |
| 268 | y1432 | | potA | |  | i-M | | spermidine/putrescine transport ATP-binding protein | 458 | |  | 5.91 | | 40541 | C | 0 |  |  | |
| 201 | y1469 | | cysA | |  | i-M | | sulfate/thiosulfate ABC transporter subunit | 336 | |  | 7.98 | | 41741 | C | 0 | + |  | |
| 37 | y1491 | | zipA | |  | i-M | | cell division protein ZipA | 167 | | 154 | 5.51 | | 37647 | IM | 1 | + |  | |
| 281 | y1524 | | yfuC | |  | i-M | | ATP-binding protein of iron ABC transporter | 63 | |  | 6.64 | | 37846 | C | 0 |  |  | |
|  | y1527 | | mltB | |  | i-M | | murein hydrolase B | 68 | |  | 8.75 | | 41342 | IM | 0 |  |  | |
|  | y1576 | | vacJ | |  | i-M | | surface lipoprotein VacJ | 63 | | 78 | 4.9 | | 28538 | IM | 0 |  |  | |
|  | y1580 | | fadJ | |  | i-M | | multifunctional fatty acid oxidation complex subunit alpha | 101 | | 313 | 9.38 | | 84129 | U | 0 | + |  | |
|  | y1603 | | dedD | | Y | i-M | | putative lipoprotein y1603 | 78 | | 61 | 7.8 | | 25637 | U | 1 |  |  | |
| 296 | y1673 | | glnQ | |  | i-M | | glutamine ABC transporter ATP-binding protein | 154 | |  | 6.46 | | 26604 | C | 0 |  |  | |
|  | y1767 | | ptsG | |  | i-M | | glucose-specific PTS system component IIBC | 114 | |  | 8.58 | | 51064 | IM | 9 |  |  | |
| 297 | y1786 | | lolD | |  | i-M | | lipoprotein ABC transporter ATP-binding subunit | 143 | | 53 | 6.56 | | 25740 | IM | 0 |  |  | |
| 210 | y1795 | | - | |  | i-M | | hypothetical protein y1795 (putative lipoprotein) | 481 | |  | 7.79 | | 22869 | OM | 0 |  |  | |
|  | y1852 | | (rssA1) | |  | i-M | | hypothetical protein y1852 (putative patatin-like phospholipase) | 53 | | 59 | 6.84 | | 45705 | IM | 1 |  |  | |
|  | y1863 | | (yebT) | |  | i-M | | hypothetical protein y1863 | 86 | |  | 6.92 | | 95966 | OM | 1 |  |  | |
| 197 | y1867 | | prc | |  | i-M | | carboxy-terminal protease for penicillin-binding protein 3 | 183 | |  | 7.6 | | 79094 | U | 1 | + |  | |
|  | y1877 | | (ampD2) | |  | i-M | | regulator (putative N-acetylmuramoyl-L-alanine amidase) | 202 | |  | 8.49 | | 31328 | C | 0 |  |  | |
|  | y1878 | | (ydjN) | |  | i-M | | symporter (predicted sodium and dicarboxylate symporter) | 189 | | 570 | 9.32 | | 49044 | IM | 9 |  |  | |
| 272 | y1896 | | yfeB | | Y | i-M | | ATP-binding protein for iron and manganese ABC transporter | 174 | |  | 6.2 | | 32490 | MSL | 0 |  |  | |
| 125 | y1919 | | (wcaG9) | |  | i-M | | bifunctional UDP glucuronic acid decarboxylase/formyl transferase | 529 | |  | 5.88 | | 75330 | U | 0 | + |  | |
| 232 | y1926 | | lplA | |  | i-M | | lipoate-protein ligase A | 114 | |  | 5.87 | | 38312 | C | 0 |  |  | |
| 233 | y1928 | | aroH | |  | i-M | | 3-deoxy-7-phosphoheptulonate synthase | 454 | |  | 5.35 | | 38628 | C | 0 |  |  | |
| 33 | y1962 | | slyB1 | |  | i-M | | outer membrane protein | 271 | | 1894 | 9.51 | | 15414 | U | 0 |  |  | |
| 234 | y1988 | | tyrR | |  | i-M | | tyrosine-binding and DNA-binding transcriptional dual regulator | 306 | |  | 5.71 | | 62791 | C | 0 |  |  | |
|  | y2003 | | hslJ | |  | i-M | | heat-inducible protein HslJ | 150 | |  | 6.27 | | 16464 | U | 0 |  |  | |
|  | y2010 | | acpD | |  | i-M | | azoreductase (acyl carrier protein phosphodiesterase) | 133 | |  | 5.02 | | 21586 | U | 0 |  |  | |
|  | y2037 | | tonB | |  | i-M | | energy transducer; part of TonB/ExbB/ExbD complex | 71 | | 326 | 6.07 | | 27485 | P | 1 |  |  | |
| 11 | y2044 | | (ompW) | |  | i-M | | outer membrane protein W | 141 | |  | 5.34 | | 23667 | OM | 0 |  |  | |
|  | y2045 | | (osmY1) | |  | i-M | | hypothetical protein y2045 (putative phospholipid-binding lipoprotein) | 111 | | 61 | 9.64 | | 11127 | P | 0 |  |  | |
| 38 | y2047 | | trpA | |  | i-M | | tryptophan synthase subunit alpha | 133 | |  | 5.84 | | 31625 | IM | 0 |  |  | |
| 207 | y2104 | | (ydgA) | | Y | i-M | | hypothetical protein y2104 (putative phospholipid-binding lipoprotein) | 1015 | | 405 | 5.26 | | 58864 | U | 1 | + |  | |
|  | y2134 | | pntB | |  | i-M | | pyridine nucleotide transhydrogenase, beta subunit | 134 | | 455 | 5.39 | | 49076 | IM | 9 |  |  | |
|  | y2135 | | pntA | |  | i-M | | NAD(P) transhydrogenase, subunit alpha | 235 | | 542 | 5.16 | | 54407 | IM | 5 |  |  | |
|  | y2160 | | sppA | |  | i-M | | protease IV (signal peptide peptidase) | 102 | | 136 | 6.69 | | 67106 | IM | 1 |  |  | |
| 252 | y2233 | | minD | |  | i-M | | cell division inhibitor MinD | 370 | | 84 | 5.06 | | 29772 | C | 0 | + |  | |
| 73 | y2234 | | minE | |  | i-M | | cell division topological specificity factor MinE | 148 | |  | 5.34 | | 10383 | C | 0 |  |  | |
|  | y2237 | | (slp) | |  | i-M | | outer membrane lipoprotein | 92 | | 55 | 9.47 | | 21996 | U | 1 |  |  | |
| 74 | y2263 | | cutC | |  | i-M | | copper homeostasis protein CutC | 352 | |  | 5.81 | | 29787 | C | 0 |  |  | |
| 84 | y2270 | | (yceM) | |  | i-M | | virulence factor | 196 | | 584 | 6.29 | | 60159 | U | 0 |  |  | |
| 259 | y2292 | | lolb | |  | i-M | | outer membrane lipoprotein LolB | 139 | | 52 | 9.16 | | 26380 | U | 0 |  |  | |
| 279 | y2315 | | - | |  | i-M | | hypothetical protein y2315 | 452 | |  | 6.06 | | 35255 | U | 0 | + |  | |
| 275 | y2364 | | (phnL4) | |  | i-M | | ABC transporter ATP-binding protein | 96 | |  | 5.82 | | 26653 | IM | 0 |  |  | |
|  | y2396 | | ybtQ | |  | i-M | | permease/ATP-binding protein of yersiniabactin-iron ABC transporter | 180 | | 409 | 6.69 | | 66593 | IM | 5 |  |  | |
|  | y2397 | | ybtP | | Y | i-M | | permease/ATP-binding protein of yersiniabactin-iron ABC transporter | 56 | | 684 | 9.05 | | 66300 | IM | 6 |  |  | |
|  | y2452 | | (efeU) | |  | i-M | | cytochrome (putative iron/lead family transporter) | 63 | |  | 6.96 | | 31205 | IM | 7 |  |  | |
|  | y2552 | | manY | |  | i-M | | mannose-specific PTS enzyme IIC | 139 | | 553 | 5.48 | | 29357 | IM | 7 |  |  | |
|  | y2553 | | manZ | |  | i-M | | mannose-specific PTS enzyme IID | 158 | | 579 | 9.42 | | 32431 | IM | 2 |  |  | |
| 200 | y2596 | | lldD | |  | i-M | | L-lactate dehydrogenase | 995 | |  | 7.82 | | 41290 | U | 0 |  |  | |
| 260 | y2633 | | ysuR | |  | i-M | | TonB-dependent outer membrane iron/siderophore receptor | 202 | | 374 | 8.81 | | 84014 | OM | 0 |  |  | |
|  | y2644 | | asmA | |  | i-M | | putative OM/lipopolysaccharide assembly protein | 123 | | 56 | 8.69 | | 68057 | U | 1 |  |  | |
| 85 | y2647 | | mrp | |  | i-M | | putative ATPase (multiple resistance and pH adaptation protein Mrp) | 213 | |  | 5.43 | | 40349 | U | 0 |  |  | |
|  | y2661 | | mglA | |  | i-M | | galactose/methyl galaxtoside transporter ATP-binding protein | 65 | |  | 7.16 | | 57084 | IM | 0 |  |  | |
| 235 | y2677 | | (uup3) | |  | i-M | | ATP-binding component of ABC transport system | 529 | | 96 | 5.27 | | 66418 | IM | 0 | + |  | |
|  | y2731 | | (yccF) | |  | i-M | | hypothetical protein y2731 (conserved inner membrane protein) | 102 | | 135 | 9.65 | | 16613 | IM | 3 |  |  | |
| 253 | y2747 | | pqiB | | Y | i-M | | paraquat-inducible protein B | 295 | | 283 | 8.68 | | 61743 | U | 1 |  |  | |
| 39 | y2750 | | uup | |  | i-M | | ABC transporter ATPase component | 377 | |  | 5.62 | | 73348 | IM | 0 | + |  | |
|  | y2765 | | mukB | |  | i-M | | condesin subunit B (cell division protein MukB) | 203 | | 157 | 5.28 | | 170261 | C | 0 |  |  | |
| 76 | y2767 | | mukF | |  | i-M | | condesin subunit F | 363 | |  | 4.82 | | 50257 | C | 0 | + |  | |
|  | y2814 | | (macA) | |  | i-M | | macrolide transporter subunit MacA | 81 | | 60 | 6.93 | | 40161 | IM | 1 |  |  | |
| 58 | y2826 | | (ybjP) | |  | i-M | | predicted lipoprotein | 348 | | 165 | 8.5 | | 20894 | U | 0 |  |  | |
| 236 | y2836 | | (smtA3) | |  | i-M | | ABC transport protein (putative iron transport) | 162 | |  | 6.19 | | 30066 | U | 0 |  |  | |
| 199 | y2864 | | dacC | |  | i-M | | D-alanyl-D-alanine carboxypeptidase (penicillin-binding protein 6) | 600 | |  | 8.86 | | 47535 | IM | 0 |  |  | |
|  | y2882 | | psaA | |  | i-M | | pH 6 antigen fimbrial subunit | 231 | |  | 5.52 | | 17909 | E | 0 |  |  | |
| 40 | y2910 | | (oppA2) | |  | i-M | | hypothetical protein y2104 (putative ABC-type oligopeptide transporter) | 1039 | |  | 7.72 | | 69129 | IM | 0 | + |  | |
| 195 | y2913 | | (yejF) | |  | i-M | | ATP-binding component of a transport system | 659 | |  | 8.26 | | 60126 | IM | 0 |  |  | |
| 261 | y2940 | | - | |  | i-M | | hypothetical protein y2940 (putative bacteriophage Mu P protein) | 194 | |  | 5.39 | | 38824 | U | 0 |  |  | |
| 41 | y2980 | | (yfaZ) | |  | i-M | | hypothetical protein y2980 | 241 | | 523 | 5.88 | | 18713 | IM | 0 |  |  | |
|  | y3018 | | (ybhL) | |  | i-M | | hypothetical protein y3018 (putative FtsH-interacting protein) | 81 | | 54 | 9.52 | | 25875 | OM | 7 |  |  | |
| 42 | y3041 | | modF | |  | i-M | | ATP-binding component of molybdate transport system | 72 | |  | 5.71 | | 58239 | IM | 0 |  |  | |
| 43 | y3058 | | tolQ | |  | i-M | | colicin uptake protein TolQ | 64 | | 75 | 6.16 | | 25348 | IM | 3 |  |  | |
| 254 | y3074 | | ppnK | |  | i-M | | inorganic polyphosphate / ATP-NAD kinase | 132 | |  | 6.04 | | 32492 | U | 0 |  |  | |
| 262 | y3076 | | smpA | |  | i-M | | small membrane protein A | 112 | | 91 | 7.79 | | 12939 | U | 0 |  |  | |
|  | y3104 | | metN | |  | i-M | | DL-methionine transporter ATP-binding subunit | 154 | |  | 6.56 | | 37681 | IM | 0 |  |  | |
| 59 | y3106 | | metQ | |  | i-M | | DL-methionine ABC transporter substrate-binding protein | 201 | | 1095 | 5.38 | | 29416 | U | 0 | + |  | |
|  | y3110 | | cutF | |  | i-M | | lipoprotein involved with copper homeostasis and adhesion | 344 | | 50 | 5.51 | | 27204 | U | 0 | + |  | |
|  | y3235 | | lamB1 | |  | i-M | | maltoporin | 85 | |  | 4.8 | | 49044 | OM | 0 |  |  | |
|  | y3242 | | - | |  | i-M | | putative maltose ABC transporter ATP-binding protein | 101 | |  | 6.15 | | 41229 | C | 0 |  |  | |
|  | y3306 | | (mscS2) | |  | i-M | | transport protein (putative mechanosensitive channel) | 99 | | 282 | 5.85 | | 31092 | IM | 3 |  |  | |
| 86 | y3311 | | (yggG) | |  | i-M | | hypothetical protein y3311 (putative Zn-dependent protease) | 259 | |  | 5.77 | | 27897 | U | 0 |  |  | |
|  | y3341 | | mltC | |  | i-M | | membrane-bound lytic murein transglycosylase C | 88 | |  | 9.15 | | 40470 | U | 0 |  |  | |
| 24 | y3385 | | iutA | |  | i-M | | TonB-dependent aerobactin outer membrane receptor | 569 | |  | 5.19 | | 80383 | OM | 0 |  |  | |
|  | y3494 | | exbD | | Y | i-M | | biopolymer transport protein ExbD of TonB-complex | 89 | | 135 | 4.6 | | 15565 | IM | 1 |  |  | |
|  | y3495 | | exbB | |  | i-M | | TonB complex protein | 166 | | 118 | 9.47 | | 37106 | IM | 4 |  |  | |
| 27 | y3560 | | cyaE | |  | i-M | | hypothetical protein y3560 (putative outer membrane efflux protein) | 232 | |  | 6.35 | | 53790 | OM | 1 |  |  | |
|  | y3586 | | - | |  | i-M | | hypothetical protein y3586 | 163 | |  | 4.88 | | 63408 | U | 0 |  |  | |
| 247 | y3622 | | ftsA | | Y | i-M | | cell division protein FtsA | 155 | |  | 5.67 | | 45797 | C | 0 | + |  | |
| 248 | y3625 | | murC | |  | i-M | | UDP-N-acetylmuramate-L-alanine ligase | 139 | |  | 5.92 | | 55628 | C | 0 |  |  | |
| 87 | y3640 | | ilvI | |  | i-M | | acetolactate synthase 3 catalytic subunit | 191 | |  | 5.87 | | 65110 | U | 0 |  |  | |
| 88 | y3656 | | hepA | |  | i-M | | ATP-dependent helicase HepA | 368 | |  | 5.06 | | 110514 | U | 0 |  |  | |
| 294 | y3658 | | - | |  | i-M | | putative type VI secretion system subunit y3658 (VasK-like component) | 63 | |  | 8.99 | | 132769 | IM | 3 |  |  | |
|  | y3659 | | - | |  | i-M | | putative type VI secretion system subunit y3659 (VasF-like component) | 57 | |  | 8.61 | | 61519 | IM | 1 |  |  | |
| 280 | y3660 | | - | |  | i-M | | hypothetical protein y3660 | 134 | |  | 5.59 | | 51448 | U | 0 |  |  | |
| 298 | y3664 | | - | |  | i-M | | putative type VI secretion system subunit y3664 | 117 | |  | 5.7 | | 42413 | U | 0 |  |  | |
| 273 | y3665 | | - | |  | i-M | | hypothetical protein y3665 | 293 | |  | 5.35 | | 84654 | U | 0 | + |  | |
| 302 | y3668 | | (vgrG5) | |  | i-M | | hypothetical protein y3668 (putative type VI secretion system subunit) | 406 | |  | 5.16 | | 85601 | U | 0 |  |  | |
| 295 | y3671 | | - | |  | i-M | | putative type VI secretion system subunit y3671 (VasA-like component) | 241 | |  | 6.25 | | 69095 | C | 0 | + |  | |
| 89 | y3674 | | - | | Y | i-M | | hypothetical protein y3674 (putative type VI secretion system subunit) | 493 | | 61 | 5.28 | | 57354 | U | 0 | + | 21.1735 | |
| 211 | y3675 | | - | | Y | i-M | | hypothetical protein y3675 (putative type VI secretion system subunit) | 426 | | 147 | 4.52 | | 21076 | C | 0 | 0.3641 | 3.0481 | |
| 290 | y3676 | | - | |  | i-M | | putative type VI secretion system subunit y3676 | 146 | |  | 4.8 | | 39103 | C | 0 |  |  | |
|  | y3731 | | (ompA5) | |  | i-M | | outer membrane lipoprotein | 63 | | 50 | 9.71 | | 18610 | OM | 1 |  |  | |
|  | y3737 | | sms | |  | i-M | | ATP-dependent protease | 144 | | 55 | 6.77 | | 49985 | U | 0 |  |  | |
|  | y3760 | | - | |  | i-M | | multidrug resistance protein (putative macrolide transporter) | 111 | | 88 | 6.62 | | 36847 | C | 0 |  |  | |
| 34 | y3803 | | (typA) | |  | i-M | | GTP-binding factor BipA/TypA | 667 | | 448 | 5.22 | | 67452 | C | 0 | 0.2620 | 16.3729 | |
|  | y3864 | | - | |  | i-M | | hypothetical protein y3864 (putative 2-component regulator subunit) | 68 | |  | 6.53 | | 94816 | IM | 2 |  |  | |
| 309 | y3891 | | glpD | | Y | i-M | | sn-glycerol-3-phosphate dehydrogenase | 116 | |  | 6.57 | | 60515 | C | 0 |  |  | |
|  | y3924 | | mrcA | |  | i-M | | peptidoglycan synthetase (penicillin-binding protein 1A) | 67 | | 68 | 7.22 | | 94748 | MSL | 1 |  |  | |
|  | y3936 | | damX | |  | i-M | | hypothetical protein y3936 (putative cell division interference protein) | 160 | | 80 | 9.34 | | 34635 | U | 1 | + |  | |
|  | y3948 | | (cirA1) | |  | i-M | | putative outer membrane receptor protein CirA | 398 | |  | 9.03 | | 75019 | OM | 1 |  |  | |
| 90 | y4020 | | trkA | |  | i-M | | potassium transporter peripheral membrane component | 140 | |  | 5.34 | | 50313 | U | 0 | + |  | |
| 256 | y4043 | | (fecB3) | |  | i-M | | solute-binding iron ABC transport protein | 303 | | 154 | 5.37 | | 35511 | P | 0 | + |  | |
| 263 | y4088 | | - | |  | i-M | | hypothetical protein y4088 (putative outer membrane lipoprotein) | 80 | | 145 | 9.16 | | 26928 | U | 1 | + |  | |
|  | y4117 | | yidC | |  | i-M | | putative inner membrane protein translocase component YidC | 75 | | 243 | 8.54 | | 61309 | IM | 5 |  |  | |
| 237 | y4128 | | pstB | |  | i-M | | ATP-binding protein of high-affinity phosphate ABC transporter | 221 | |  | 5.62 | | 29381 | MSL | 0 |  |  | |
|  | y4141 | | atpB | |  | i-M | | F0F1 ATP synthase subunit A | 104 | | 121 | 6.29 | | 30391 | IM | 5 |  |  | |
|  | y4144 | | gidA | |  | i-M | | glucose-inhibited division protein A | 126 | |  | 6.4 | | 73325 | U | 0 |  |  | |
| 196 | YPKMT066 | | caf1A | |  | i-M | | fimbrial usher protein of F1 capsule antigen | 583 | |  | 9.13 | | 93401 | OM | 0 |  |  | |
| 282 | Y1020 | |  | |  | i-M | | hypothetical protein Y1020 | 91 | |  | 8.52 | | 14970 | U | 0 |  |  | |
| 1 | y0032 | | lamB | | Y | i-OM | | maltoporin | 751 | | 586 | 6.02 | | 53392 | OM | 0 |  |  | |
| 32 | y0221 | | slyB | |  | i-OM | | outer membrane receptor | 390 | | 2621 | 11.12 | | 15504 | U | 0 |  |  | |
| 4 | y0325 | | btuB | |  | i-OM | | vitamin B12/cobalamin outer membrane transporter | 672 | | 1203 | 5.28 | | 73080 | OM | 0 |  |  | |
| 5 | y0396 | | pldA | |  | i-OM | | outer membrane phospholipase A | 376 | | 240 | 5.03 | | 33738 | OM | 0 |  |  | |
| 6 | y0543 | | hmuR | |  | i-OM | | TonB-dependent outer membrane receptor (hemin uptake) | 1064 | | 1673 | 5.06 | | 74241 | OM | 0 | + |  | |
| 213 | y0659 | | - | |  | i-OM | | hypothetical protein y0659 ( putative outer membrane protein) | 1011 | | 618 | 6.37 | | 64480 | OM | 0 |  |  | |
| 7 | y0850 | | (cirA2) | |  | i-OM | | TonB-dependent outer membrane receptor (iron transport) | 669 | | 1847 | 6.38 | | 80093 | OM | 0 | + |  | |
| 8 | y1324 | | ail | | Y | i-OM | | adhesion and invasion locus protein Ail | 1145 | |  | 8.51 | | 21612 | OM | 1 | + |  | |
| 56 | y1419 | | nlpB | | Y | i-OM | | lipoprotein | 647 | | 199 | 5.85 | | 38492 | U | 0 | + |  | |
| 9 | y1577 | | fadL | |  | i-OM | | long-chain fatty acid outer membrane transporter | 1008 | | 1833 | 4.82 | | 45670 | OM | 0 | + |  | |
| 10 | y1682 | | ompX | | Y | i-OM | | outer membrane protein X | 487 | | 2879 | 5.85 | | 18951 | OM | 1 |  | 60.6915 | |
| 12 | y2167 | | mipA | | Y | i-OM | | hypothetical protein y2167 (outer membrane protein V) | 643 | | 95 | 6.12 | | 28967 | OM | 1 | + |  | |
| 57 | y2358 | | hmsF | |  | i-OM | | hemin storage system, biofilm formation protein HmsF | 476 | | 254 | 5.61 | | 76722 | U | 0 | + | 11.8444 | |
| 13 | y2359 | | hmsH | |  | i-OM | | hemin storage system, biofilm formation protein HmsH | 1087 | | 1103 | 5.38 | | 95915 | OM | 0 | + | 36.5814 | |
| 14 | y2404 | | psn | | Y | i-OM | | pesticin/yersiniabactin outer membrane receptor | 2801 | | 11208 | 5.54 | | 73850 | OM | 0 | + | 30.7921 | |
| 15 | y2556 | | fcuA | |  | i-OM | | TonB-dependent outer membrane ferrichrome receptor | 801 | | 1019 | 6.75 | | 84153 | OM | 0 |  |  | |
| 16 | y2735 | | ompA | | Y | i-OM | | outer membrane protein A | 2001 | | 14008 | 8.63 | | 39363 | OM | 1 | + | 35.8417 | |
| 17 | y2759 | | ompF | | Y | i-OM | | outer membrane porin protein OmpF | 870 | | 3501 | 4.71 | | 41266 | OM | 1 |  | 50.4722 | |
| 18 | y2872 | | yiuR | |  | i-OM | | outer membrane iron/siderophore receptor | 947 | | 1138 | 5.94 | | 73978 | OM | 0 | + |  | |
| 19 | y2966 | | ompC | | Y | i-OM | | outer membrane porin protein OmpC | 1770 | | 624 | 4.83 | | 41383 | OM | 0 |  | 71.1749 | |
| 20 | y2983 | | phoE | |  | i-OM | | outer membrane porin protein PhoE | 874 | | 3444 | 5.53 | | 40664 | OM | 0 |  | 69.1345 | |
| 21 | y3054 | | pal | |  | i-OM | | peptidoglycan-associated outer membrane lipoprotein | 548 | | 3690 | 6.3 | | 18130 | OM | 0 | + |  | |
| 22 | y3127 | | (yaeT) | | Y | i-OM | | outer membrane protein assembly factor YaeT | 2226 | | 1795 | 5.42 | | 87784 | OM | 1 | + |  | |
| 23 | y3343 | | - | | Y | i-OM | | TonB-dependent outer membrane receptor (ferrichrome-iron transport) | 1042 | | 437 | 6.05 | | 83079 | OM | 1 | + |  | |
| 208 | y3404 | | - | |  | i-OM | | TonB-dependent outer membrane receptor | 1390 | | 467 | 5.29 | | 79066 | OM | 0 |  |  | |
| 25 | y3480 | | papC | |  | i-OM | | outer membrane usher protein (P-pilus assembly protein) | 178 | |  | 5.01 | | 90429 | OM | 0 |  |  | |
| 26 | y3516 | | tolC | | Y | i-OM | | outer membrane channel protein (efflux system component TolC) | 1120 | | 2362 | 7.75 | | 50902 | OM | 0 | + |  | |
| 28 | y3680 | | imp | |  | i-OM | | organic solvent tolerance protein | 598 | | 779 | 7.08 | | 89405 | OM | 0 |  |  | |
| 206 | y4083 | | - | |  | i-OM | | hypothetical protein y4083 (putative secreted protein) | 219 | |  | 5.91 | | 17449 | U | 1 | + |  | |
| 31 | YPKp07 | | pla | |  | i-OM | | plasminogen activator/outer membrane protease | 1792 | | 854 | 5.9 | | 35798 | OM | 0 | + |  | |
| 204 | YP_2181 | | lpp | |  | i-OM | | major outer membrane lipoprotein | 496 | |  | 8.93 | | 8727 | OM | 0 |  |  | |
| 285 | y0037 | | - | |  | p-M | | putative type VI secretion system subunit y0037 | 331 | |  | 4.97 | | 18510 | U | 0 | + | 0.5303 | |
| 188 | y0067 | | cpxR | |  | p-M | | DNA response regulator in two-component regulatory system with CpxA | 195 | |  | 5.47 | | 27507 | C | 0 | 0.0261 |  | |
| 301 | y0084 | | rfaF | |  | p-M | | ADP-heptose-LPS heptosyltransferase II | 157 | |  | 6.47 | | 40245 | U | 0 |  | 0.3252 | |
| 128 | y0098 | | (yicC) | | Y | p-M | | hypothetical protein y0098 (putative stress-induced protein) | 471 | |  | 5.16 | | 33379 | C | 0 | + |  | |
| 113 | y0137 | | degQ | | Y | p-M | | periplasmic serine endoprotease | 884 | | 75 | 8.51 | | 48149 | P | 1 | + | 0.0869 | |
| 315 | y0163 | | (yjgF) | |  | p-M | | similar to E. coli ketoacid-binding protein YjgF | 480 | |  | 9.1 | | 15157 | U | 0 | + |  | |
| 117 | y0194 | | - | |  | p-M | | putative carbon-nitrogen hydrolase | 544 | | 52 | 7.83 | | 32424 | U | 0 | + |  | |
| 276 | y0391 | | - | |  | p-M | | putative ABC transporter ATP-binding protein | 82 | | 91 | 5.04 | | 44013 | C | 0 | 0.0185 | 2.2788 | |
| 186 | y0542 | | hmuS | |  | p-M | | hemin uptake system component | 156 | |  | 5.51 | | 39124 | U | 0 | + |  | |
| 183 | y0546 | | - | |  | p-M | | orfX protein in hemin uptake locus (putative heme iron utilization protein) | 115 | |  | 5.86 | | 20245 | U | 0 | + | 0.8166 | |
| 152 | y0556 | | terA | |  | p-M | | tellurium resistance protein | 261 | |  | 6.02 | | 42794 | U | 0 | + | 2.9956 | |
| 118 | y0596 | | - | |  | p-M | | hypothetical protein y0596 | 114 | |  | 8.67 | | 16086 | U | 0 |  |  | |
|  | y0606 | | aspA | |  | p-M | | aspartate ammonia-lyase (aspartase) | 982 | |  | 5.26 | | 53318 | C | 0 | + | 1.2846 | |
| 299 | y0616 | | frdB | |  | p-M | | fumarate reductase iron-sulfur subunit | 418 | |  | 6.59 | | 28098 | C | 0 |  | 1.1795 | |
| 243 | y0617 | | frdA | |  | p-M | | fumarate reductase flavoprotein subunit | 350 | |  | 5.87 | | 67421 | P | 0 | 0.2065 | 0.0141 | |
| 170 | y0686 | | (yhbC) | |  | p-M | | hypothetical protein y0686 | 171 | |  | 4.63 | | 17074 | C | 0 |  |  | |
| 63 | y0696 | | deaD | |  | p-M | | inducible ATP-independent RNA helicase | 371 | |  | 9.1 | | 74039 | C | 0 | + | 1.4993 | |
| 229 | y0722 | | phnM | |  | p-M | | phosphonate metabolism protein | 179 | |  | 5.7 | | 41257 | C | 0 |  |  | |
| 317 | y0733 | | nrdD | |  | p-M | | anaerobic ribonucleoside triphosphate reductase | 668 | |  | 6.21 | | 80901 | C | 0 |  | 0.9527 | |
| 189 | y0766 | | pdhR | |  | p-M | | transcriptional regulator of pyruvate dehydrogenase complex | 112 | |  | 6.1 | | 32115 | C | 0 |  |  | |
| 134 | y0767 | | aceE | | Y | p-M | | pyruvate dehydrogenase subunit E1 (dihycarboxylase component) | 920 | | 220 | 5.47 | | 99838 | C | 0 | 0.0166 | 2.3342 | |
| 91 | y0768 | | aceF | | Y | p-M | | dihydrolipoamide acetyltransferase (pyruvate dehydrogenase complex) | 818 | | 1310 | 5.32 | | 53972 | C | 0 | 0.0421 |  | |
| 92 | y0769 | | lpdA | | Y | p-M | | dihydrolipoamide dehydrogenase (pyruvate dehydrogenase complex) | 776 | |  | 5.74 | | 50919 | C | 0 | 0.0441 | 0.1150 | |
| 223 | y0818 | | aph | | Y | p-M | | sulfite reductase (NADPH), flavoprotein beta subunit | 448 | | 57 | 5.17 | | 69032 | C | 0 | 0.0533 |  | |
| 120 | y0870 | | katY | |  | p-M | | catalase (hydroperoxidase HPI(I)) KatG/KatY | 793 | |  | 7.17 | | 83063 | U | 0 | 0.1795 | 3.3660 | |
| 137 | y0914 | | clpB | |  | p-M | | protein disaggregation chaperone | 1740 | | 90 | 5.51 | | 96430 | C | 0 | + | 0.4791 | |
| 218 | y0964 | | frsA | |  | p-M | | hypothetical protein y0964 (putative fermentation/respiration switch protein) | 154 | | 73 | 5.88 | | 48008 | U | 0 | 0.0288 |  | |
| 219 | y0966 | | proB | |  | p-M | | gamma-glutamyl kinase | 331 | |  | 6.13 | | 39381 | C | 0 | + | 0.5548 | |
| 66 | y0978 | | phoB | |  | p-M | | positive response regulator for pho regulon | 224 | |  | 5.47 | | 27502 | C | 0 |  |  | |
|  | y1043 | | tesB | |  | p-M | | acyl-CoA thioesterase II | 594 | |  | 5.94 | | 33431 | C | 0 | 0.1029 |  | |
| 140 | y1064 | | htpG | | Y | p-M | | heat shock protein 90 | 1936 | | 360 | 5.03 | | 70987 | C | 0 | 0.0874 | 3.6624 | |
| 244 | y1194 | | (phoH1) | |  | p-M | | ATP-binding protein in pho regulon | 230 | |  | 5.92 | | 41907 | C | 0 |  | 0.4924 | |
| 220 | y1195 | | (yleE) | |  | p-M | | hypothetical protein y1195 (putative iron-sulfur protein and methylthiolase) | 178 | |  | 5.41 | | 58203 | U | 0 | + |  | |
| 313 | y1237 | | ureA | |  | p-M | | urease (urea amidohydrolase) gamma subunit | 199 | |  | 5.35 | | 11042 | U | 0 | + | 0.3832 | |
| 94 | y1240 | | ureE | |  | p-M | | urease accessory protein UreE | 248 | |  | 5.83 | | 26405 | C | 0 | + | 0.5765 | |
| 291 | y1242 | | ureG | |  | p-M | | urease accessory protein UreG | 336 | |  | 4.88 | | 24106 | C | 0 | + |  | |
| 278 | y1290 | | rpoE | |  | p-M | | RNA polymerase sigma factor RpoE | 410 | |  | 5.08 | | 21860 | U | 0 |  |  | |
| 68 | y1295 | | lepA | |  | p-M | | GTP-binding protein LepA | 337 | |  | 5.57 | | 67238 | C | 0 | + |  | |
|  | y1298 | | era | |  | p-M | | GTP-binding protein Era | 78 | |  | 6.57 | | 34565 | C | 0 |  |  | |
| 82 | y1357 | | engA | |  | p-M | | GTP-binding protein EngA (putative ferrous iron uptake family protein) | 445 | |  | 5.47 | | 55029 | U | 0 |  | 0.6268 | |
| 164 | y1362 | | guaB | | Y | p-M | | inositol-5-monophosphate dehydrogenase | 325 | | 671 | 6.05 | | 55310 | C | 1 | 0.1711 | 0.3147 | |
| 163 | y1397 | | ppx | |  | p-M | | exopolyphosphatase | 302 | | 205 | 5.89 | | 58903 | U | 0 | + | 0.3021 | |
| 314 | y1455 | | (ygiW) | |  | p-M | | putative outer membrane protein | 662 | |  | 7.82 | | 14927 | U | 0 | 0.4237 | 0.2037 | |
| 95 | y1485 | | crr | |  | p-M | | glucose-specific PTS system enzyme IIA subunit | 485 | |  | 4.8 | | 18227 | C | 0 | 0.0525 | 1.1676 | |
| 193 | y1579 | | fadI | |  | p-M | | 3-ketoacyl-CoA thiolase | 1021 | | 67 | 6.68 | | 46396 | C | 0 |  |  | |
| 165 | y1601 | | accD | |  | p-M | | acetyl-CoA carboxylase beta subunit | 105 | | 241 | 8.48 | | 39483 | U | 0 |  |  | |
|  | y1611 | | (yfcH) | |  | p-M | | putative sugar nucleotide epimerase | 633 | |  | 5.7 | | 33852 | U | 0 |  |  | |
|  | y1631 | | nuoB | |  | p-M | | NADH dehydrogenase subunit B | 96 | |  | 5.76 | | 25880 | U | 0 | + | 0.5218 | |
| 71 | y1632 | | nuoC | | Y | p-M | | binfunctional NADH:ubiquinone oxidoreductase subunit C/D | 654 | |  | 5.83 | | 69070 | C | 0 |  | 0.6327 | |
| 97 | y1633 | | nuoE | |  | p-M | | NADH dehydrogenase subunit E | 128 | |  | 5 | | 20920 | C | 0 | + | 0.4171 | |
| 251 | y1634 | | nuoF | |  | p-M | | NADH dehydrogenase I chain F | 187 | |  | 6.3 | | 50934 | C | 0 | + | 0.6256 | |
| 83 | y1635 | | nuoG | | Y | p-M | | NADH dehydrogenase subunit G | 300 | |  | 5.72 | | 101584 | U | 0 | + | 0.5636 | |
| 277 | y1637 | | nuoI | |  | p-M | | NADH dehydrogenase subunit I | 122 | |  | 5.55 | | 21058 | C | 0 |  | 0.1907 | |
| 124 | y1677 | | dps | |  | p-M | | DNA protection during starvation conditions | 503 | | 80 | 5.87 | | 18859 | U | 0 | 0.0808 | 0.1992 | |
| 72 | y1794 | | phoP | |  | p-M | | DNA response regulator in two-comp. regulatory system with PhoQ | 464 | |  | 5.51 | | 25625 | C | 0 | + | 1.2623 | |
| 205 | y1845 | | - | | Y | p-M | | probable N-acetylmuramoyl-L-alanine amidase | 353 | | 96 | 5.52 | | 28780 | C | 0 | 0.0523 | 2.1489 | |
|  | y1935 | | sufB | | Y | p-M | | cysteine desulfurase activator complex subunit SufB | 124 | | 63 | 4.77 | | 55548 | U | 0 | + |  | |
| 292 | y1936 | | sufC | |  | p-M | | cysteine desulfurase ATPase component | 164 | |  | 5.25 | | 28618 | MSL | 0 | + |  | |
| 98 | y1980 | | pspA | |  | p-M | | phage shock protein A | 653 | |  | 5.77 | | 25386 | C | 0 |  | 0.1394 | |
| 99 | y2023 | | adhE | | Y | p-M | | CoA-linked acetaldehyde dehydrogenase | 922 | | 171 | 6.39 | | 97040 | C | 0 | + | 0.5072 | |
| 143 | y2168 | | (yeaG) | |  | p-M | | hypothetical protein y2168 (putative Ser protein kinase) | 651 | |  | 5.66 | | 74433 | C | 0 | 0.1064 | 6.5885 | |
| 177 | y2295 | | prsA | |  | p-M | | ribose-phosphate pyrophosphokinase | 486 | |  | 5.26 | | 35118 | C | 0 | 0.0232 | 0.1637 | |
| 214 | y2402 | | ybtT | |  | p-M | | yersiniabactin thioesterase | 232 | | 229 | 5.68 | | 24794 | U | 0 | + |  | |
| 75 | y2455 | | putA | | Y | p-M | | proline dehydrogenase, P5C dehydrogenase | 889 | | 481 | 5.76 | | 145005 | C | 0 | + | 0.4771 | |
| 101 | y2551 | | manX | | Y | p-M | | mannose-specific PTS enzyme IIAB | 1253 | | 220 | 5.58 | | 35165 | C | 0 | 1.1958 | 0.5624 | |
| 156 | y2739 | | (lonB) | | Y | p-M | | ATP-dependent protease (Lon protease family) | 157 | |  | 4.74 | | 66259 | U | 0 | + | 0.3972 | |
| 102 | y2766 | | mukE | |  | p-M | | condesin subunit E | 74 | |  | 4.88 | | 28863 | C | 0 | + | 1.3700 | |
| 77 | y2809 | | clpA | | Y | p-M | | ATP-binding component of serine protease with AAA-type ATPase (F) | 671 | | 144 | 5.78 | | 84751 | C | 0 | + | 3.6637 | |
| 182 | y3065 | | sucC | | Y | p-M | | succinyl-CoA synthetase subunit beta | 181 | | 136 | 5.27 | | 41665 | C | 0 | 0.0055 | 0.3748 | |
| 104 | y3066 | | sucB | | Y | p-M | | dihydrolipoamide acetyltransferase (subunit of 2-oxoglutarate DH complex) | 296 | | 269 | 5.48 | | 44286 | C | 0 | 0.0247 | 0.2025 | |
| 222 | y3068 | | sdhB | |  | p-M | | succinate dehydrogenase iron-sulfur subunit | 735 | |  | 6.87 | | 27264 | U | 0 |  | 2.5492 | |
| 150 | y3069 | | sdhA | | Y | p-M | | succinate dehydrogenase flavoprotein subunit | 1424 | |  | 5.68 | | 64834 | MSL | 0 | 0.1571 | 0.6931 | |
| 166 | y3123 | | lpxA | |  | p-M | | UDP-N-acetylglucosamine acyltransferase | 183 | |  | 6.36 | | 28499 | C | 0 | + |  | |
| 316 | y3124 | | fabZ | |  | p-M | | (3R)-hydroxymyristoyl ACP dehydratase | 473 | |  | 6.1 | | 19732 | C | 0 | 0.0523 | 0.1189 | |
| 265 | y3126 | | ompH | |  | p-M | | periplasmic chaperone (automembrane protein H) | 159 | | 120 | 9.47 | | 21630 | OM | 0 | + |  | |
|  | y3219 | | agaV | |  | p-M | | PTS family enzyme IIB component 2 (EIIB-AGA) | 78 | |  | 6.96 | | 18213 | C | 0 |  |  | |
| 202 | y3295 | | visC | |  | p-M | | hypothetical protein y3295 (putative octaprenyl methoxyphenyl hydrolase) | 226 | |  | 7.8 | | 53425 | IM | 0 | + |  | |
| 312 | y3555 | | aspC | |  | p-M | | aspartate aminotransferase | 959 | |  | 5.13 | | 45665 | C | 0 | 0.0327 | 4.1008 | |
| 246 | y3590 | | - | |  | p-M | | enzyme (predicted methyltransferase) | 201 | | 145 | 5.43 | | 44400 | C | 0 | + |  | |
| 175 | y3609 | | - | |  | p-M | | hypothetical protein y3609 (putative inner membrane protein) | 166 | | 131 | 6.1 | | 10991 | C | 1 |  | 1.4766 | |
| 44 | y3617 | | secA | | Y | p-M | | preprotein translocase subunit SecA of Sec membrane complex | 1149 | | 193 | 5.29 | | 102788 | IM | 0 | 1.1938 | 2.9481 | |
| 45 | y3621 | | ftsZ | | Y | p-M | | cell division protein FtsZ | 912 | | 162 | 4.75 | | 40400 | IM | 0 | + | 6.0321 | |
|  | y3657 | | - | |  | p-M | | hypothetical protein y3657 | 194 | |  | 5.1 | | 13489 | U | 0 |  | 0.3308 | |
| 78 | y3669 | | (clpB2) | | Y | p-M | | ATP-dependent protease associated with type VI secretion system | 552 | | 137 | 5.61 | | 96900 | C | 0 | + | 4.2459 | |
| 167 | y3753 | | rsmC | |  | p-M | | 16S ribosomal RNA m2G1207 methyltransferase | 274 | |  | 6.12 | | 37947 | U | 0 |  |  | |
|  | y3773 | | (lsrF) | |  | p-M | | putative autoinducer-2 (AI-2) aldolase | 1050 | |  | 6.08 | | 31819 | U | 0 | 0.0602 | 0.5109 | |
| 107 | y3859 | | uspA | |  | p-M | | universal stress protein | 474 | |  | 4.77 | | 16485 | C | 0 | 1.2800 | 2.0239 | |
| 79 | y3916 | | ompR | | Y | p-M | | osmolarity response regulator OmpR | 548 | |  | 6.16 | | 27408 | C | 0 | 0.1880 | 5.8967 | |
|  | y4101 | | ibpB | |  | p-M | | heat shock chaperone IbpB | 400 | |  | 5.23 | | 19141 | U | 0 | 0.2585 | 0.6073 | |
| 80 | y4127 | | phoU | |  | p-M | | transcriptional regulator PhoU | 524 | |  | 5.4 | | 27704 | C | 0 | + |  | |
| 168 | y4133 | | glmU | |  | p-M | | GlcNAc-1-phosph. uridyltransferase/glucosamine-1-phosph. acetyltransferase | 285 | |  | 6.02 | | 49282 | C | 0 | 0.1504 | 1.6928 | |
| 109 | y4134 | | atpC | | Y | p-M | | F0F1 ATP synthase subunit epsilon | 347 | |  | 6.08 | | 15107 | C | 0 | + | 2.3829 | |
| 110 | y4135 | | atpD | | Y | p-M | | F0F1 ATP synthase subunit beta | 1623 | | 338 | 4.95 | | 50210 | C | 0 | 0.3737 | 0.6371 | |
| 293 | y4136 | | atpG | |  | p-M | | F0F1 ATP synthase subunit gamma | 131 | |  | 8.99 | | 31671 | U | 0 | + |  | |
| 127 | y4137 | | atpA | | Y | p-M | | F0F1 ATP synthase subunit alpha | 937 | | 369 | 5.52 | | 55337 | U | 0 | 0.2860 | 0.5453 | |
| 111 | y4138 | | atpH | |  | p-M | | F0F1 ATP synthase subunit delta | 333 | |  | 5.31 | | 19597 | C | 0 | + | 0.5574 | |
| 112 | y4139 | | atpF | |  | p-M | | F0F1 ATP synthase subunit B | 320 | | 476 | 5.42 | | 17304 | C | 1 |  | 0.0918 | |
| 29 | YPKMT065 | | caf1 | |  | p-M | | F1 capsule antigen | 266 | |  | 4.83 | | 17655 | OM | 0 | + |  | |
|  | YPKMT089 | | parA | |  | p-M | | partitioning protein | 161 | |  | 5.85 | | 44929 | C | 0 | + |  | |
| 129 | y0294 | | hslU | |  | p-M* | | ATP-dependent protease ATP-binding subunit | 349 | |  | 5.25 | | 49887 | C | 0 | + |  | |
| 132 | y0609 | | groEL | | Y | p-M* | | chaperonin GroEL | 1341 | | 2597 | 4.91 | | 57509 | C | 0 | 0.0368 | 0.3197 | |
| 255 | y0651 | | fklB | |  | p-M* | | FKBP-type peptidyl-prolyl cis-trans isomerase | 155 | | 132 | 4.71 | | 22412 | OM | 0 | 0.0334 | 0.3262 | |
| 159 | y0687 | | nusA | | Y | p-M* | | transcription elongation factor NusA | 346 | | 67 | 4.52 | | 55549 | C | 0 | 0.0483 |  | |
| 310 | y0694 | | pnp | | Y | p-M* | | polynucleotide phosphorylase/polyadenylase | 804 | | 240 | 5.33 | | 79856 | C | 0 | 0.0170 | 0.1285 | |
| 114 | y0807 | | htrA | |  | p-M* | | periplasmic serine endoprotease Do | 543 | |  | 8.81 | | 49950 | P | 1 | + |  | |
| 115 | y0815 | | sodC | |  | p-M* | | periplasmic superoxide dismutase precursor (Cu-Zn) | 282 | |  | 7.85 | | 20126 | P | 0 | + |  | |
| 93 | y0988 | | ahpC | |  | p-M* | | peroxidase (putative alkyl hydroperoxide reductase subunit C) | 501 | | 268 | 5.7 | | 22358 | C | 0 | 0.0076 | 0.4757 | |
| 121 | y1026 | | tig | |  | p-M* | | trigger factor | 568 | | 124 | 4.85 | | 48211 | U | 0 | 0.0038 | 2.6369 | |
| 139 | y1029 | | lon | | Y | p-M* | | DNA-binding ATP-dependent protease La (heat shock protein K) | 881 | | 66 | 5.86 | | 89749 | C | 0 | 0.0600 | 1.4952 | |
| 122 | y1239 | | ureC | |  | p-M* | | urease (urea amidohydrolase) alpha subunit | 567 | | 175 | 5.41 | | 58838 | U | 0 | 0.0354 | 0.2079 | |
| 311 | y1363 | | guaA | |  | p-M* | | bifunctional GMP synthase/glutamine amidotransferase protein | 557 | | 205 | 5.55 | | 58841 | C | 0 | 0.0193 |  | |
| 154 | y1620 | | pta | |  | p-M* | | phosphate acetyltransferase | 742 | | 103 | 5.22 | | 77753 | U | 0 | 0.0314 | 2.2748 | |
| 144 | y2394 | | ybtS | |  | p-M* | | salicylate synthase Irp9 | 1021 | | 136 | 5.66 | | 48414 | C | 0 | 0.3643 | 5.1016 | |
| 221 | y2399 | | irp2 | |  | p-M* | | HMWP2 nonribosomal peptide synthetase | 393 | | 1413 | 5.78 | | 230218 | MSL | 0 | + |  | |
| 155 | y2400 | | irp1 | |  | p-M* | | HMWP1 nonribosomal peptide synthetase | 737 | | 2448 | 5.43 | | 351658 | U | 0 | + |  | |
|  | y2401 | | ybtU | |  | p-M* | | thiazolinyl-S-HMWP1 reductase | 483 | | 134 | 6.39 | | 43680 | U | 0 | + |  | |
| 145 | y2403 | | ybtE | |  | p-M* | | salicyl-AMP ligase | 430 | | 390 | 5.32 | | 57701 | C | 0 | 0.0732 |  | |
| 116 | y3055 | | tolB | |  | p-M* | | periplasmic translocation protein TolB | 796 | | 128 | 6.61 | | 46016 | P | 0 | + | 0.5991 | |
| 105 | y3067 | | kgd | | Y | p-M* | | alpha-ketoglutarate decarboxylase (subunit of 2-oxoglutarate DH complex) | 771 | | 247 | 5.96 | | 105614 | C | 0 | 0.0075 | 0.4970 | |
| 304 | y3314 | | metK | | Y | p-M* | | S-adenosylmethionine synthetase | 414 | | 116 | 5.36 | | 42221 | C | 0 | 0.0269 | 15.1072 | |
| 172 | y3673 | | - | | Y | p-M* | | hypothetical protein y3673 (putative HCP subunit of type VI secretion system) | 192 | | 154 | 5.18 | | 19000 | U | 0 | 0.0097 | 5.6729 | |
| 157 | y3706 | | dnaK | | Y | p-M* | | molecular chaperone DnaK | 1413 | | 136 | 4.86 | | 69040 | U | 0 | 0.0116 | 2.5737 | |
| 148 | y3985 | | fusA | | Y | p-M* | | GTP-binding protein chain elongation factor EF-G | 1041 | | 2600 | 5.29 | | 77716 | C | 0 | 0.0368 | 4.4743 | |
| 108 | y3986 | | tufB | | Y | p-M* | | elongation factor Tu | 1854 | | 3226 | 5.17 | | 43304 | C | 0 | 0.0294 | 3.5536 | |
| 160 | y4015 | | rpoA | | Y | p-M* | | DNA-directed RNA polymerase alpha subunit | 484 | | 155 | 4.98 | | 36714 | C | 0 | 0.0335 | 0.3033 | |
| 30 | YPKMT067 | | caf1M | |  | p-M* | | periplasmic chaperone for F1 capsule antigen | 503 | |  | 8.38 | | 28847 | OM | 0 | + |  | |
| 158 | Y1069 | | ymt | | Y | p-M* | | murine toxin | 646 | | 64 | 5.6 | | 67675 | U | 0 | 0.0055 | 0.2065 | |
| 169 | y0483 | | rplL | |  | C/R | | 50S ribosomal subunit protein L7/L12 | 302 | | 123 | 4.47 | | 12523 | U | 0 | 0.0320 | 0.0680 | |
| 173 | y0645 | | rpsF | | Y | C/R | | 30S ribosomal protein S6 | 280 | | 461 | 5.34 | | 14998 | C | 0 | 0.0697 | 1.5600 | |
| 176 | y0647 | | rplI | | Y | C/R | | 50S ribosomal protein L9 | 611 | | 213 | 6.3 | | 15852 | C | 0 | 0.0978 | 0.1840 | |
| 138 | y0960 | | pepD | |  | C/R | | aminoacyl-histidine dipeptidase (peptidase D) | 423 | |  | 5.45 | | 53187 | C | 0 | 0.0199 | 0.6176 | |
| 178 | y1489 | | cysK | |  | C/R | | cysteine synthase A | 909 | | 413 | 5.25 | | 34354 | U | 0 | 0.0063 | 0.4972 | |
| 269 | y1990 | | tpx | |  | C/R | | thiol peroxidase | 226 | | 107 | 6.7 | | 20848 | U | 0 | 0.0071 | 1.1603 | |
| 191 | y2165 | | gapA | |  | C/R | | glyceraldehyde-3-phosphate dehydrogenase | 636 | | 859 | 6.19 | | 36105 | C | 0 | 0.0063 | 0.4473 | |
| 100 | y2524 | | ftn | |  | C/R | | cytoplasmic ferritin | 269 | | 81 | 5.62 | | 23613 | C | 1 | 0.0101 |  | |
| 103 | y2781 | | rpsA | | Y | C/R | | 30S ribosomal protein S1 | 1296 | | 64 | 4.96 | | 61362 | C | 0 | 0.0902 | 0.5269 | |
| 179 | y3307 | | fba | |  | C/R | | fructose-bisphosphate aldolase | 428 | | 74 | 5.71 | | 39332 | U | 0 | 0.0077 | 0.6531 | |
| 162 | y3310 | | tktA | |  | C/R | | transketolase | 1423 | | 574 | 5.8 | | 72113 | C | 1 | 0.0067 | 0.3000 | |
| 271 | y0028 | | malE | |  | P | | periplasmic maltose-binding protein | 509 | |  | 6.18 | | 43802 | P | 0 | + |  | |
|  |  | |  | |  |  | |  |  | |  |  | |  |  |  |  |  | |
| **Part II. Proteins analyzed in 2D-LC-MS/MS experiments** | | | | | | | | | | | | | | | | |  | | |
|  | **Locus tag** | **Gene name** | |  | |  | **Protein name and description** | | |  | **MS score** | | **pI** | **Mr** | **P-SORTb** | **TMHMM** |  | | |
|  | y0046 | glpF | |  | |  | facilitator for glycerol uptake | | |  | 121 | | 5.52 | 29766 | IM | 6 |  | | |
|  | y0074 | secB | |  | |  | protein export; molecular chaperone | | |  | 106 | | 4.09 | 17793 | C | 0 |  | | |
|  | y0076 | - | |  | |  | hypothetical protein y0076 | | |  | 160 | | 9.37 | 15916 | C | 1 |  | | |
|  | y0106 | gltS | |  | |  | sodium/glutamate symporter | | |  | 222 | | 8.23 | 42697 | IM | 11 |  | | |
|  | y0139 | degS | |  | |  | serine endoprotease | | |  | 127 | | 6.12 | 38349 | P | 1 |  | | |
|  | y0144 | (yrbD) | |  | |  | ATP-binding protein of putative ABC superfamily transporter y0144 | | |  | 287 | | 5.33 | 20289 | U | 1 |  | | |
|  | y0166 | treB | |  | |  | PTS system enzyme IIBC | | |  | 63 | | 9.01 | 52713 | IM | 11 |  | | |
|  | y0195 | - | |  | |  | hypothetical protein y0195 | | |  | 73 | | 5.75 | 116479 | OM | 0 |  | | |
|  | y0239 | (yjcD) | |  | |  | putative symporter for nucleobases/cations | | |  | 61 | | 5.82 | 46218 | IM | 13 |  | | |
|  | y0296 | ftsN | |  | |  | essential cell division protein | | |  | 99 | | 10.19 | 30525 | IM | 1 |  | | |
|  | y0300 | (yiaA) | |  | |  | hypothetical protein y0300 | | |  | 74 | | 6.23 | 17037 | IM | 4 |  | | |
|  | y0372 | wecF | |  | |  | TDP-Fuc4NAc:lipidII transferase | | |  | 61 | | 9.4 | 51619 | IM | 11 |  | | |
|  | y0384 | (clcA) | |  | |  | hypothetical protein y0384, putative outer membrane lipoprotein | | |  | 403 | | 6.07 | 6622 | U | 0 |  | | |
|  | y0413 | - | |  | |  | putative inner membrane protein y0413 | | |  | 68 | | 9.47 | 15862 | U | 1 |  | | |
|  | y0437 | ugpC | |  | |  | ATP-binding component of n-glycerol-3-phosphate ABC transporter | | |  | 60 | | 7.11 | 39461 | C | 1 |  | | |
|  | y0451 | ubiB | |  | |  | putative ubiquinone biosynthesis protein UbiB | | |  | 149 | | 6.86 | 62388 | IM | 1 |  | | |
|  | y0452 | tatA | |  | |  | Sec-independent protein translocase protein TatA | | |  | 206 | | 6.56 | 9383 | U | 1 |  | | |
|  | y0454 | tatC | |  | |  | TatABCE protein translocation system subunit | | |  | 85 | | 7.74 | 28706 | IM | 6 |  | | |
|  | y0511 | gltP | |  | |  | glutamate/aspartate:proton symporter | | |  | 90 | | 8.92 | 47022 | IM | 12 |  | | |
|  | y0558 | terC | |  | |  | putative tellurium resistance protein | | |  | 242 | | 6.75 | 35248 | IM | 8 |  | | |
|  | y0570 | plsB | |  | |  | glycerol-3-phosphate acyltransferase | | |  | 55 | | 9.33 | 99096 | U | 1 |  | | |
|  | y0630 | hfq | |  | |  | putative host factor I for bacteriophage Q beta replication | | |  | 193 | | 6.9 | 11124 | U | 0 |  | | |
|  | y0800 | - | |  | |  | chloride channel protein | | |  | 275 | | 9.34 | 51040 | IM | 11 |  | | |
|  | y0946 | fadE | |  | |  | putative acyl-CoA dehydrogenase | | |  | 137 | | 8.42 | 88912 | IM | 6 |  | | |
|  | y0953 | nqrC | |  | |  | Na-translocating NADH ubiquinone oxidoreductase, gamma chain | | |  | 63 | | 9.28 | 31201 | U | 2 |  | | |
|  | y0956 | nqrF | |  | |  | Na-translocating NADH ubiquinone oxidoreductase, beta chain | | |  | 102 | | 4.95 | 45426 | C | 1 |  | | |
|  | y0994 | secF | |  | |  | protein secretion, membrane protein | | |  | 352 | | 5.2 | 37463 | IM | 6 |  | | |
|  | y1017 | - | |  | |  | major facilitator family transport protein | | |  | 87 | | 9.3 | 48758 | IM | 12 |  | | |
|  | y1020 | cyoB | |  | |  | cytochrome o ubiquinol oxidase subunit I | | |  | 56 | | 6.6 | 74067 | IM | 14 |  | | |
|  | y1044 | - | |  | |  | glycoprotein/polysaccharide metabolism | | |  | 125 | | 6.71 | 17903 | U | 0 |  | | |
|  | y1066 | hemH | |  | |  | ferrochelatase | | |  | 52 | | 7.1 | 36390 | C | 1 |  | | |
|  | y1086 | rosB | |  | |  | putative potassium efflux protein | | |  | 135 | | 5.24 | 60459 | IM | 13 |  | | |
|  | y1093 | - | |  | |  | putative copper exporting ATPase | | |  | 253 | | 5.3 | 101733 | IM | 9 |  | | |
|  | y1096 | (ybbK) | |  | |  | putative protease | | |  | 230 | | 5.49 | 33468 | U | 1 |  | | |
|  | y1101 | (ybbP) | |  | |  | putative oxidoreductase / ABC transporter permease | | |  | 73 | | 9.55 | 90078 | IM | 10 |  | | |
|  | y1120 | - | |  | |  | predicted Zn-dependent hydrolases of the beta-lactamase fold | | |  | 52 | | 8.66 | 15464 | U | 2 |  | | |
|  | y1143 | - | |  | |  | conserved hypothetical protein | | |  | 66 | | 5.71 | 223992 | MSL | 2 |  | | |
|  | y1190 | lnt | |  | |  | apolipoprotein N-acyltransferase | | |  | 123 | | 9.31 | 59323 | IM | 8 |  | | |
|  | y1264 | kdpB | |  | |  | ATPase of high-affinity potassium transport system, B chain | | |  | 62 | | 6.71 | 72916 | IM | 7 |  | | |
|  | y1296 | lepB | |  | |  | leader peptidase (signal peptidase I) | | |  | 55 | | 6.9 | 36811 | IM | 2 |  | | |
|  | y1325 | hcaT | |  | |  | MFS (major facilitator superfamily) transporter | | |  | 118 | | 9.8 | 42436 | IM | 12 |  | | |
|  | y1352 | - | |  | |  | paral putative membrane protein | | |  | 259 | | 6.32 | 36418 | U | 1 |  | | |
|  | y1395 | - | |  | |  | putative divalent cation (magnesium) transporter | | |  | 192 | | 5.14 | 54207 | IM | 5 |  | | |
|  | y1399 | (pstA1) | |  | |  | putative inner membrane permease of high-affinity phosphate ABC transporter | | |  | 122 | | 8.99 | 81288 | IM | 10 |  | | |
|  | y1499 | nupC | |  | |  | permease of transport system for 3 nucleosides | | |  | 69 | | 8.73 | 42509 | IM | 9 |  | | |
|  | y1525 | yfuB | |  | |  | inner membrane permease of iron ABC transporter | | |  | 92 | | 9.74 | 57103 | IM | 12 |  | | |
|  | y1630 | nuoA | |  | |  | NADH dehydrogenase I chain A | | |  | 292 | | 9.46 | 18365 | IM | 3 |  | | |
|  | y1639 | nuoK | |  | |  | NADH dehydrogenase I chain K | | |  | 103 | | 6.7 | 10873 | IM | 3 |  | | |
|  | y1641 | nuoM | |  | |  | NADH dehydrogenase I chain M | | |  | 60 | | 9.04 | 56788 | IM | 15 |  | | |
|  | y1771 | - | |  | |  | putative lipoprotein y1771 | | |  | 60 | | 9.36 | 16342 | U | 1 |  | | |
|  | y1785 | lolC | |  | |  | outer membrane-specific lipoprotein transporter subunit LolC | | |  | 51 | | 9.12 | 43105 | IM | 4 |  | | |
|  | y1823 | motA | |  | |  | flagellar motor protein MotA | | |  | 89 | | 5.48 | 32044 | IM | 4 |  | | |
|  | y1891 | yfeE | |  | |  | putative regulator of yfeABCD | | |  | 117 | | 9.3 | 20899 | IM | 3 |  | | |
|  | y1894 | yfeD | |  | |  | permease for iron and manganese ABC transporter | | |  | 237 | | 8.62 | 32181 | IM | 8 |  | | |
|  | y1895 | yfeC | |  | |  | permease for iron and manganese ABC transporter | | |  | 115 | | 8.44 | 31786 | IM | 8 |  | | |
|  | y1918 | - | |  | |  | undecaprenyl phosphate 4-deoxy-4-formamido-L-arabinose transferase | | |  | 97 | | 6.08 | 36398 | IM | 2 |  | | |
|  | y1945 | - | |  | |  | multidrug efflux protein | | |  | 51 | | 9.68 | 49369 | IM | 12 |  | | |
|  | y1971 | tppB | |  | |  | putative tripeptide transporter permease | | |  | 58 | | 9.02 | 56980 | IM | 14 |  | | |
|  | y1982 | pspC | |  | |  | phage shock protein | | |  | 74 | | 7.93 | 13455 | U | 1 |  | | |
|  | y1985 | (ycjF) | |  | |  | conserved inner membrane protein | | |  | 69 | | 9.02 | 39240 | IM | 4 |  | | |
|  | y1994 | zntB | |  | |  | Zinc transport protein zntB | | |  | 64 | | 5.07 | 37791 | U | 2 |  | | |
|  | y2042 | - | |  | |  | hypothetical protein y2042 | | |  | 89 | | 10.53 | 27736 | IM | 6 |  | | |
|  | y2071 | osmB | |  | |  | osmotically inducible lipoprotein B | | |  | 77 | | 11.01 | 6809 | U | 2 |  | | |
|  | y2232 | minC | |  | |  | cell division inhibitor | | |  | 89 | | 7.7 | 24759 | U | 1 |  | | |
|  | y2247 | msbB | |  | |  | lipid A biosynthesis (KDO)2-(lauroyl)-lipid IVA acyltransferase | | |  | 91 | | 8.61 | 37064 | IM | 1 |  | | |
|  | y2352 | chaA | |  | |  | sodium-calcium/proton antiporter | | |  | 60 | | 6.92 | 39127 | IM | 11 |  | | |
|  | y2356 | hmsS | |  | |  | inner membrane hemin storage locus protein HmsS | | |  | 104 | | 6.18 | 17491 | C | 2 |  | | |
|  | y2357 | (pgaC) | |  | |  | glycosyltransferase, probably involved in cell wall biogenesis | | |  | 124 | | 9.68 | 51996 | IM | 5 |  | | |
|  | y2367 | - | |  | |  | putative integral membrane protein | | |  | 101 | | 8.5 | 53936 | IM | 8 |  | | |
|  | y2370 | - | |  | |  | putative integral membrane protein | | |  | 243 | | 5.24 | 71311 | IM | 8 |  | | |
|  | y2371 | - | |  | |  | putative transport protein of MFS family | | |  | 243 | | 9.83 | 57777 | IM | 14 |  | | |
|  | y2374 | ansP | |  | |  | L-asparagine permease | | |  | 82 | | 9.1 | 55288 | IM | 12 |  | | |
|  | y2447 | cycA | |  | |  | symporter of D-alanine, D-serine, and glycine | | |  | 123 | | 9.48 | 52278 | IM | 12 |  | | |
|  | y2449 | wrbA | |  | |  | trp repressor binding protein | | |  | 118 | | 7.07 | 21477 | IM | 3 |  | | |
|  | y2453 | putP | |  | |  | major sodium/proline symporter | | |  | 57 | | 8.94 | 53533 | IM | 13 |  | | |
|  | y2458 | - | |  | |  | putative ATP-binding component of glutamate/aspartate ABC transporter | | |  | 81 | | 8.91 | 27972 | IM | 0 |  | | |
|  | y2540 | hpaX | |  | |  | hypothetical 4-hydroxyphenylacetate permease | | |  | 58 | | 9.65 | 49973 | IM | 12 |  | | |
|  | y2612 | (yeeF) | |  | |  | amino acid permease | | |  | 70 | | 8.93 | 50150 | IM | 12 |  | | |
|  | y2615 | - | |  | |  | ribose ABC transporter permease | | |  | 85 | | 9.46 | 34904 | IM | 10 |  | | |
|  | y2640 | ysuI | |  | |  | putative siderophore biosynthetic protein | | |  | 69 | | 6.11 | 50784 | U | 1 |  | | |
|  | y2654 | - | |  | |  | hypothetical protein y2654 | | |  | 112 | | 9.2 | 16779 | IM | 4 |  | | |
|  | y2777 | msbA | |  | |  | ATP-binding transport protein; multicopy suppressor of htrB | | |  | 59 | | 7.16 | 64592 | IM | 5 |  | | |
|  | y2789 | focA | |  | |  | probable formate transporter | | |  | 50 | | 7.04 | 30999 | IM | 6 |  | | |
|  | y2800 | ftsK | |  | |  | cell division protein | | |  | 61 | | 4.79 | 140879 | OM | 4 |  | | |
|  | y2801 | lrp | |  | |  | leucine-responsive transcriptional regulator | | |  | 174 | | 8.56 | 18939 | C | 1 |  | | |
|  | y2857 | - | |  | |  | putative transport protein | | |  | 60 | | 8.75 | 60191 | IM | 11 |  | | |
|  | y2862 | sdaC | |  | |  | probable serine transporter | | |  | 198 | | 9.23 | 48363 | IM | 10 |  | | |
|  | y2885 | fruA | |  | |  | PTS system, fructose-specific IIBC transport protein | | |  | 134 | | 8.81 | 57748 | IM | 10 |  | | |
|  | y2907 | (yeiU) | |  | |  | putative inner membrane protein y2907 | | |  | 57 | | 9.69 | 26147 | IM | 5 |  | | |
|  | y2911 | (yejB) | |  | |  | putative transport system permease protein | | |  | 261 | | 8.61 | 40611 | IM | 6 |  | | |
|  | y2912 | - | |  | |  | putative transport system permease protein | | |  | 247 | | 9.54 | 38133 | IM | 6 |  | | |
|  | y2924 | - | |  | |  | putative sulfatase | | |  | 63 | | 6.13 | 67432 | IM | 5 |  | | |
|  | y2939 | - | |  | |  | hypothetical protein y2939 | | |  | 53 | | 6.04 | 21200 | U | 0 |  | | |
|  | y2946 | - | |  | |  | putative bacteriophage tail sheath protein | | |  | 58 | | 5.53 | 53458 | U | 0 |  | | |
|  | y2957 | ompT | |  | |  | outer membrane protein 3b (a), protease VII | | |  | 105 | | 5.97 | 34863 | OM | 0 |  | | |
|  | y3004 | - | |  | |  | putative inner membrane permease of phosphonate ABC transporter | | |  | 196 | | 9.99 | 33421 | IM | 5 |  | | |
|  | y3005 | - | |  | |  | putative inner membrane permease of phosphonate ABC transporter | | |  | 147 | | 9.52 | 32108 | IM | 4 |  | | |
|  | y3007 | phnC | |  | |  | ATP-binding component of phosphonate transport | | |  | 95 | | 8.82 | 32469 | IM | 0 |  | | |
|  | y3057 | tolR | |  | |  | colicin uptake protein TolR | | |  | 109 | | 5.66 | 15500 | IM | 1 |  | | |
|  | y3063 | cydA | |  | |  | cytochrome d terminal oxidase, polypeptide subunit I | | |  | 270 | | 6.04 | 58231 | IM | 9 |  | | |
|  | y3128 | (yaeL) | |  | |  | Zinc metallopeptidase | | |  | 392 | | 6.93 | 49398 | IM | 4 |  | | |
|  | y3172 | lgt | |  | |  | phosphatidylglycerol-prolipoprotein diacylglyceryl transferase | | |  | 118 | | 9.48 | 32787 | IM | 7 |  | | |
|  | y3278 | creD | |  | |  | inner membrane protein | | |  | 118 | | 6.51 | 56065 | IM | 6 |  | | |
|  | y3329 | - | |  | |  | putative resistance protein | | |  | 73 | | 9.34 | 20795 | IM | 4 |  | | |
|  | y3344 | - | |  | |  | putative permease of ribose ABC transporter | | |  | 51 | | 9.49 | 35855 | IM | 10 |  | | |
|  | y3396 | yapH | |  | |  | putative autotransporter adhesin | | |  | 59 | | 4.11 | 370936 | MSL | 0 |  | | |
|  | y3506 | plsC | |  | |  | 1-acyl-sn-glycerol-3-phosphate acyltransferase | | |  | 71 | | 9.66 | 27188 | U | 2 |  | | |
|  | y3532 | (plsY) | |  | |  | putative glycerol-3-phosphate acyltransferase PlsY | | |  | 266 | | 9.39 | 26764 | IM | 5 |  | | |
|  | y3554 | - | |  | |  | putative sodium ion:proton antiporter | | |  | 107 | | 8.48 | 51214 | IM | 12 |  | | |
|  | y3588 | hdeD | |  | |  | acid-resistance membrane protein | | |  | 58 | | 9.51 | 20492 | IM | 6 |  | | |
|  | y3595 | - | |  | |  | Na/dicarboxylate symporter | | |  | 59 | | 6.95 | 45546 | IM | 9 |  | | |
|  | y3626 | murG | |  | |  | UDP-N-acetylglucosamine:N-acetylmuramyl-(pentapeptide) pyrophosphoryl-undecaprenol N-acetylglucosamine transferase | | |  | 160 | | 9.72 | 37750 | IM | 2 |  | | |
|  | y3707 | (yaaH) | |  | |  | putative inner membrane protein y3707 | | |  | 358 | | 4.92 | 21647 | IM | 5 |  | | |
|  | y3709 | - | |  | |  | putative proline/betaine transporter | | |  | 54 | | 9.4 | 50471 | IM | 12 |  | | |
|  | y3728 | - | |  | |  | putative cation transport protein | | |  | 66 | | 5.69 | 99150 | IM | 10 |  | | |
|  | y3759 | mexB | |  | |  | putative multidrug resistance protein | | |  | 555 | | 5.17 | 113317 | IM | 12 |  | | |
|  | y3762 | - | |  | |  | hypothetical protein y3762 | | |  | 150 | | 9.34 | 44879 | IM | 11 |  | | |
|  | y3801 | rbn | |  | |  | tRNA processing exoribonuclease BN | | |  | 65 | | 9.24 | 35434 | IM | 7 |  | | |
|  | y3823 | - | |  | |  | hypothetical protein y3823 | | |  | 109 | | 6.61 | 48925 | OM | 1 |  | | |
|  | y3898 | glpG | |  | |  | intramembrane serine protease GlpG | | |  | 68 | | 6.64 | 31285 | IM | 6 |  | | |
|  | y3911 | feoB | |  | |  | ferrous iron transport protein B | | |  | 84 | | 5.94 | 84745 | IM | 11 |  | | |
|  | y4010 | prlA | |  | |  | preprotein translocase subunit SecY | | |  | 137 | | 9.89 | 48599 | IM | 10 |  | | |
|  | y4035 | (pldB3) | |  | |  | putative membrane protein | | |  | 198 | | 8.94 | 54025 | IM | 1 |  | | |
|  | y4093 | (yiaD) | |  | |  | putative outer membrane protein | | |  | 231 | | 9.71 | 22219 | OM | 3 |  | | |
|  | y4103 | - | |  | |  | hypothetical protein y4103 | | |  | 111 | | 4.72 | 12317 | U | 1 |  | | |
|  | y4129 | pstA | |  | |  | inner membrane permease of high-affinity phosphate ABC transporter | | |  | 109 | | 10.14 | 32311 | IM | 6 |  | | |
|  | y4130 | pstC | |  | |  | phosphate transporter permease subunit | | |  | 68 | | 6.62 | 34886 | IM | 6 |  | | |
|  | y4140 | atpE | |  | |  | membrane-bound ATP synthase, F0 sector, subunit c | | |  | 63 | | 4.44 | 8250 | IM | 2 |  | | |
|  | YKCD1p18 | lcrF | |  | |  | transcriptional regulator VirF | | |  | 53 | | 9.06 | 30813 | U | 0 |  | | |

| **a)** protein spot numbers match those displayed in Figures 1, 5 and Supplemental Figures (Additional Files 2-9); |
| --- |
| **b)** locus tags from *Yersinia pestis* KIM genome annotations; |
| **c)** gene names from KIM genome annotations (databases in NCBI and ERIC: [www.ericbrc.org/portal/eric](http://www.ericbrc.org/portal/eric) were used); gene names listed in parentheses are from orthologs in *E. coli* K12 or *Y. pestis* Microtus 91001 strains, if sequence identity score was at least 70%; |
| **d)** proteins detected in high M_r_ size exclusion chromatography fractions (>250 kDa) derived from hpH-MBR membrane extracts (Y = observed); MPC: abbreviation for multi-subunit protein complex; |
| **e)** subcellular localization and membrane attachement of proteins based on differential 2D gel display data: i-M: integral membrane proteins and less water-soluble peripheral membrane proteins; i-OM: integral and lipid-anchored outer membrane proteins; p-M: more water-soluble peripheral membrane proteins; p-M*: tentative peripheral membrane proteins with high abundance in soluble fractions; C/R: cytoplasmic/ribosomal membrane-contaminating proteins; |
| **f)** information derived from *Y. pestis* KIM genome annotations or orthologs in *E. coli* K12 or *Y. pestis* Microtus 91001; |
| **g)** Mascot score: highest score obtained from MS-analyzed spots in 2D gels (by MALDI-TOFTOF or nESI-LC-MS/MS); |
| **h)** MS score obtained from 2D-LC-MS/MS analysis experiments; |
| **i)** pI and M_r_ values of proteins calculated from polypeptide sequences annotated in the KIM genome; |
| **j)** subcellular localization data according to PSORTb search results; abbreviations: C, cytoplasm, P: periplasm, MSL: multiple cellular localizations, IM: inner membrane, OM: outer membrane, U: unknown; |
| **k)** number of inner membrane α-helical transmembrane domains in proteins predicted by the algorithm TMHMM; |
| **l)** enrichment factor for a protein in all membrane *vs.* CYP fractions (E_M_); equation is listed in the 'Method' section; blank space: value missing when X_N_ (geometric means) was not determined in membrane and/or CYP fractions; symbol '+' indicates that a membrane-associated protein was identified by MS in PPS or CYP fractions; |
| **m)** enrichment factor for a protein in hpH-MBR and usb-MBR *vs.* hs-MBR fractions (E_IM_); see other notes under l); |
