# Supplementary figures and images for: Integral and peripheral association of proteins and protein complexes with Yersinia pestis inner and outer membranes
Source: Proteome Sci. 2009 Feb 19;7:5. doi: 10.1186/1477-5956-7-5 (PMC2663777; doi:10.1186/1477-5956-7-5)

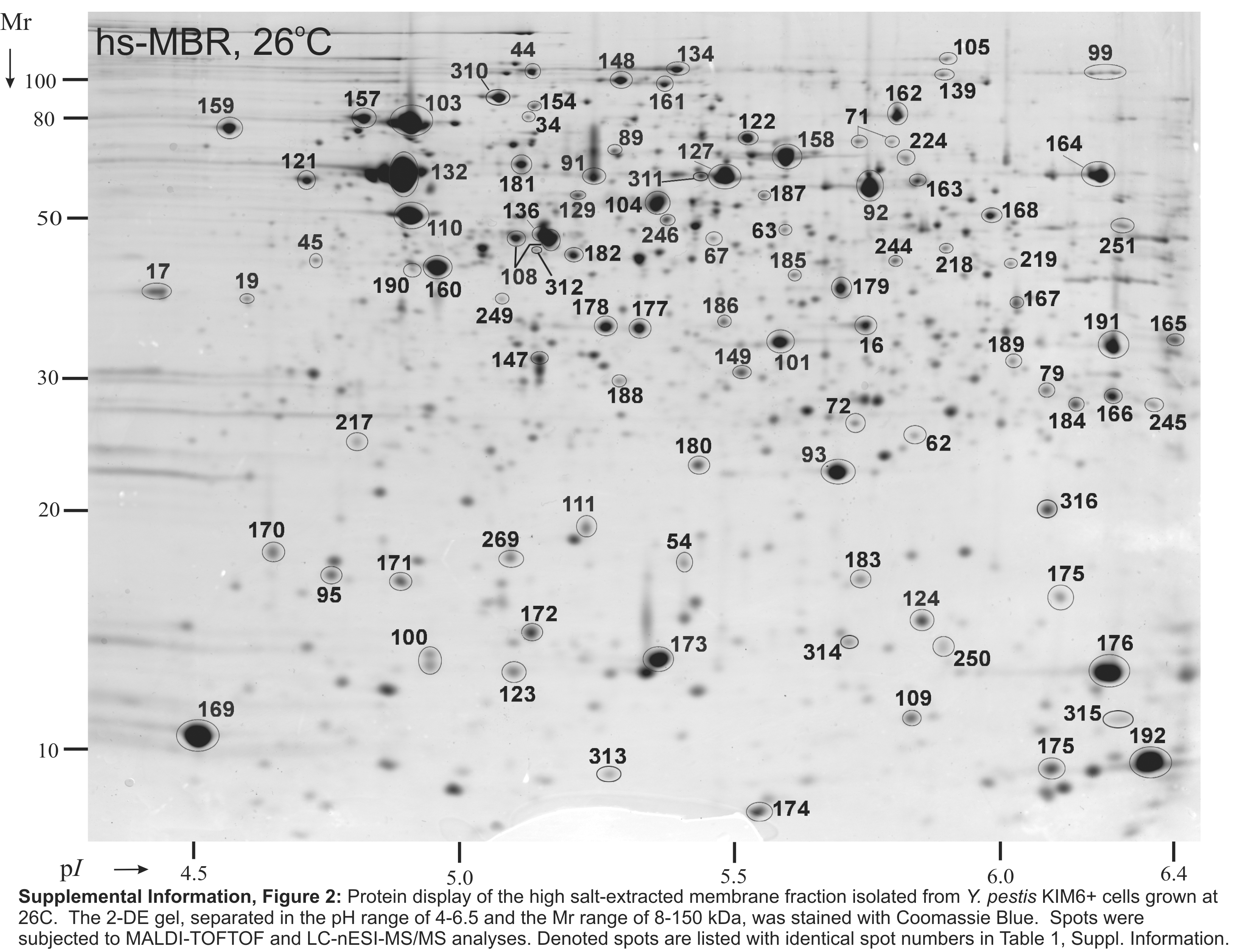

Supplement: Additional file 3 — Supplemental Information (Figure Two). Protein display of the high salt-extracted membrane fraction isolated from Y. pestis KIM6+ cells grown at 26°C (pH range of 4–6.5). [file 1477-5956-7-5-S3.tiff]

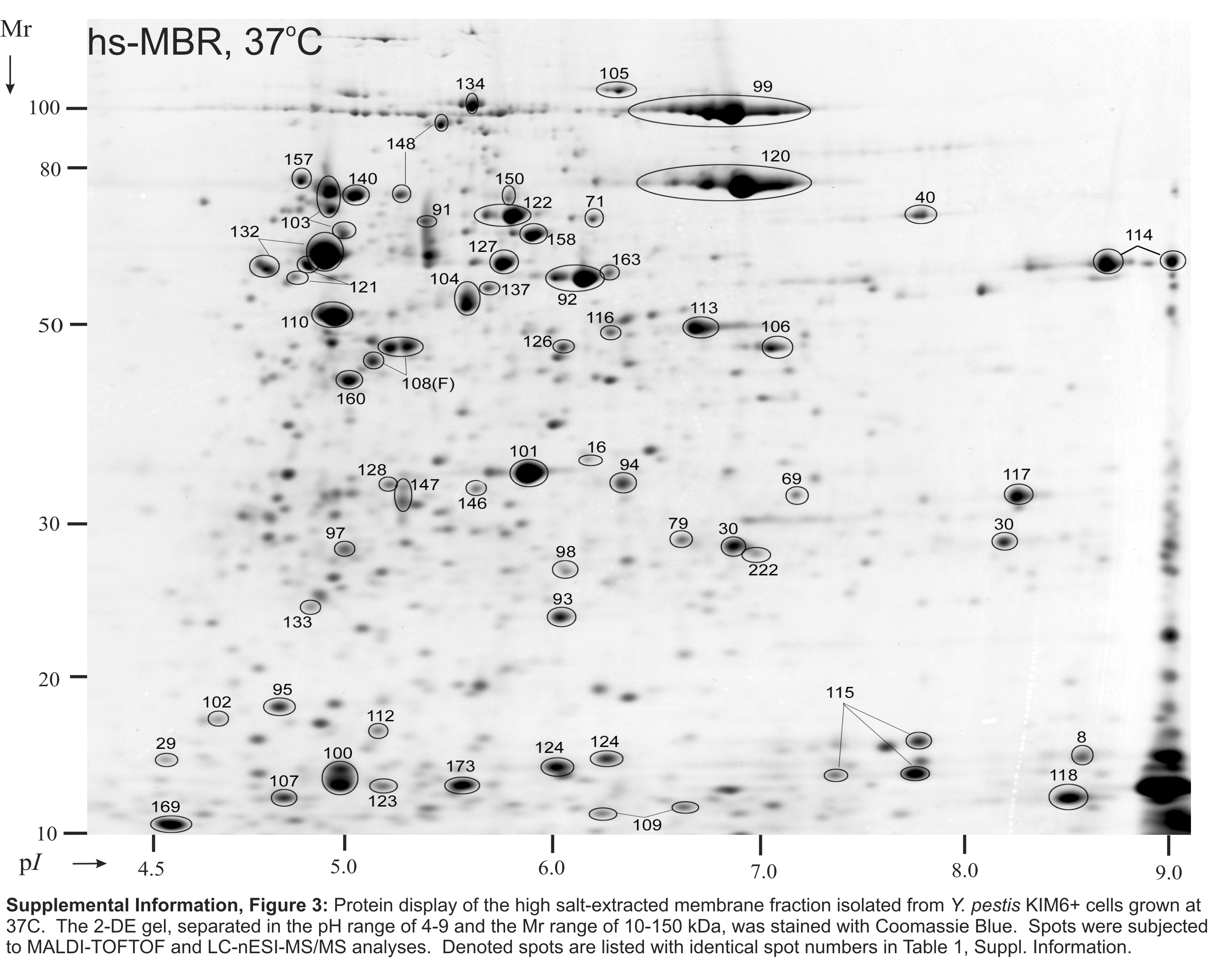

Supplement: Additional file 4 — Supplemental Information (Figure Three). Protein display of the high salt-extracted membrane fraction isolated from Y. pestis KIM6+ cells grown at 37°C (pH range of 4–9). [file 1477-5956-7-5-S4.tiff]

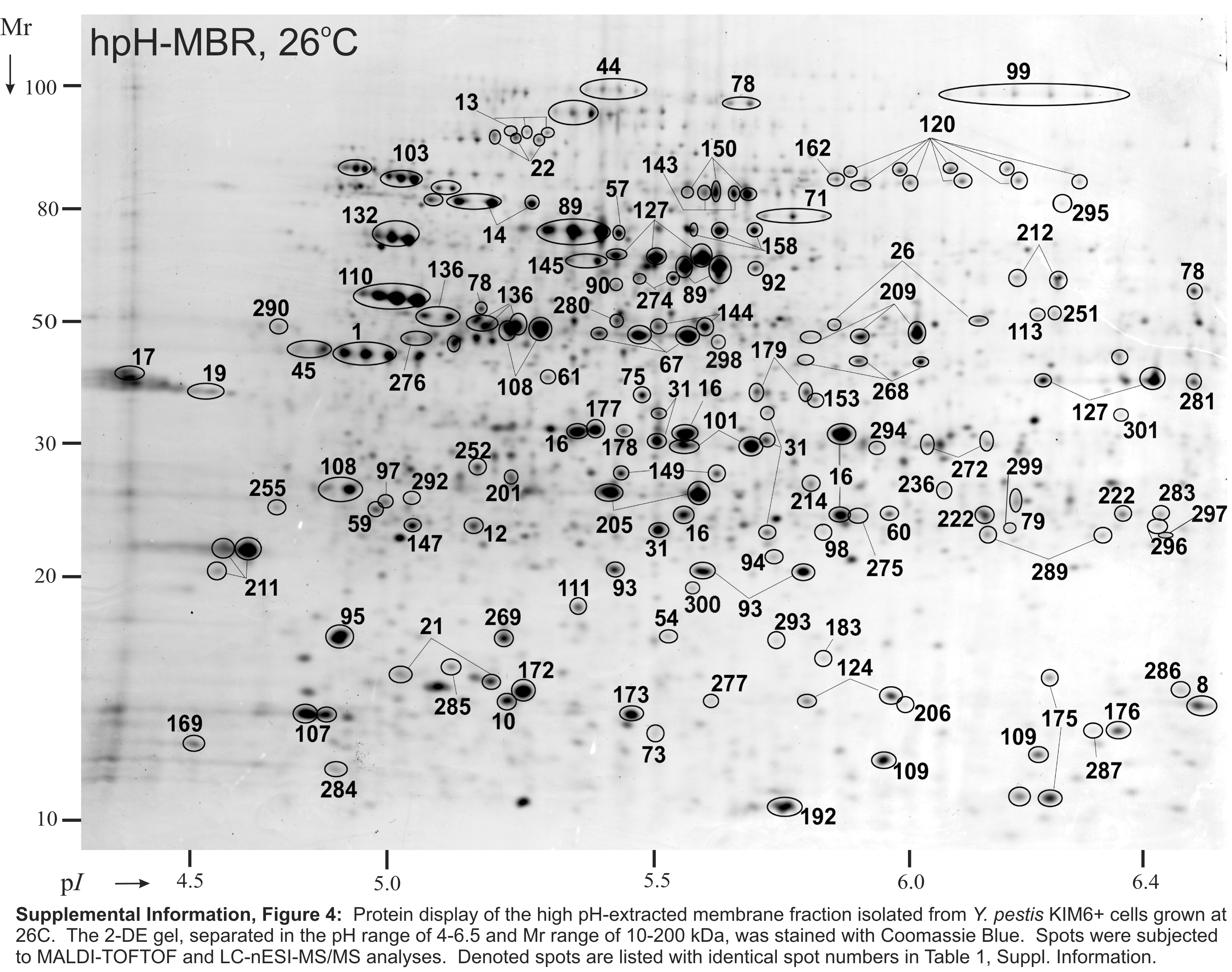

Supplement: Additional file 5 — Supplemental Information (Figure Four). Protein display of the high pH-extracted membrane fraction isolated from Y. pestis KIM6+ cells grown at 26°C (pH range of 4–6.5). [file 1477-5956-7-5-S5.tiff]

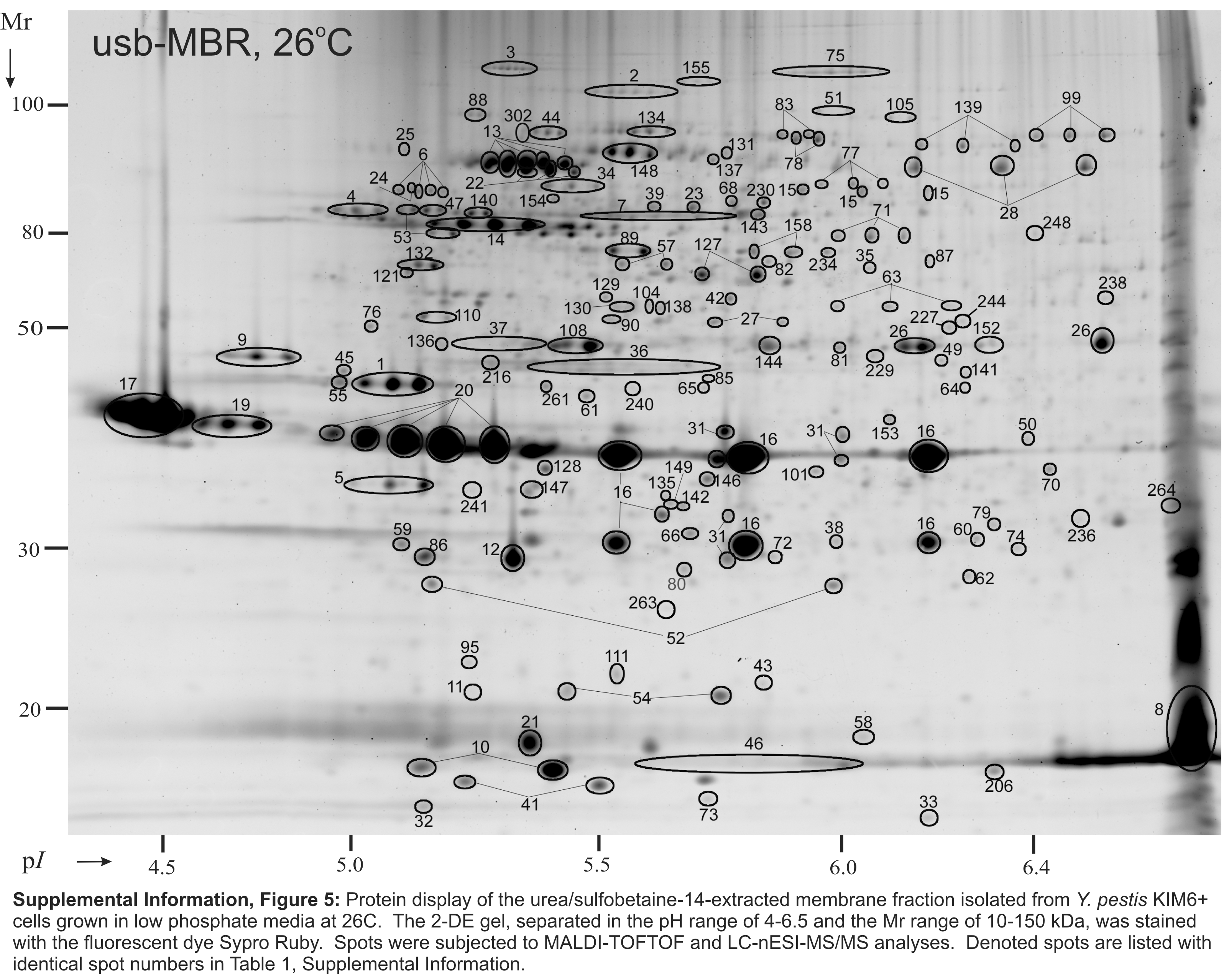

Supplement: Additional file 6 — Supplemental Information (Figure Five). Protein display of urea/sulfobetaine-14-extracted membrane fraction isolated from Y. pestis KIM6+ cells grown in low phosphate media at 26°C (pH range of 4–6.5). [file 1477-5956-7-5-S6.tiff]

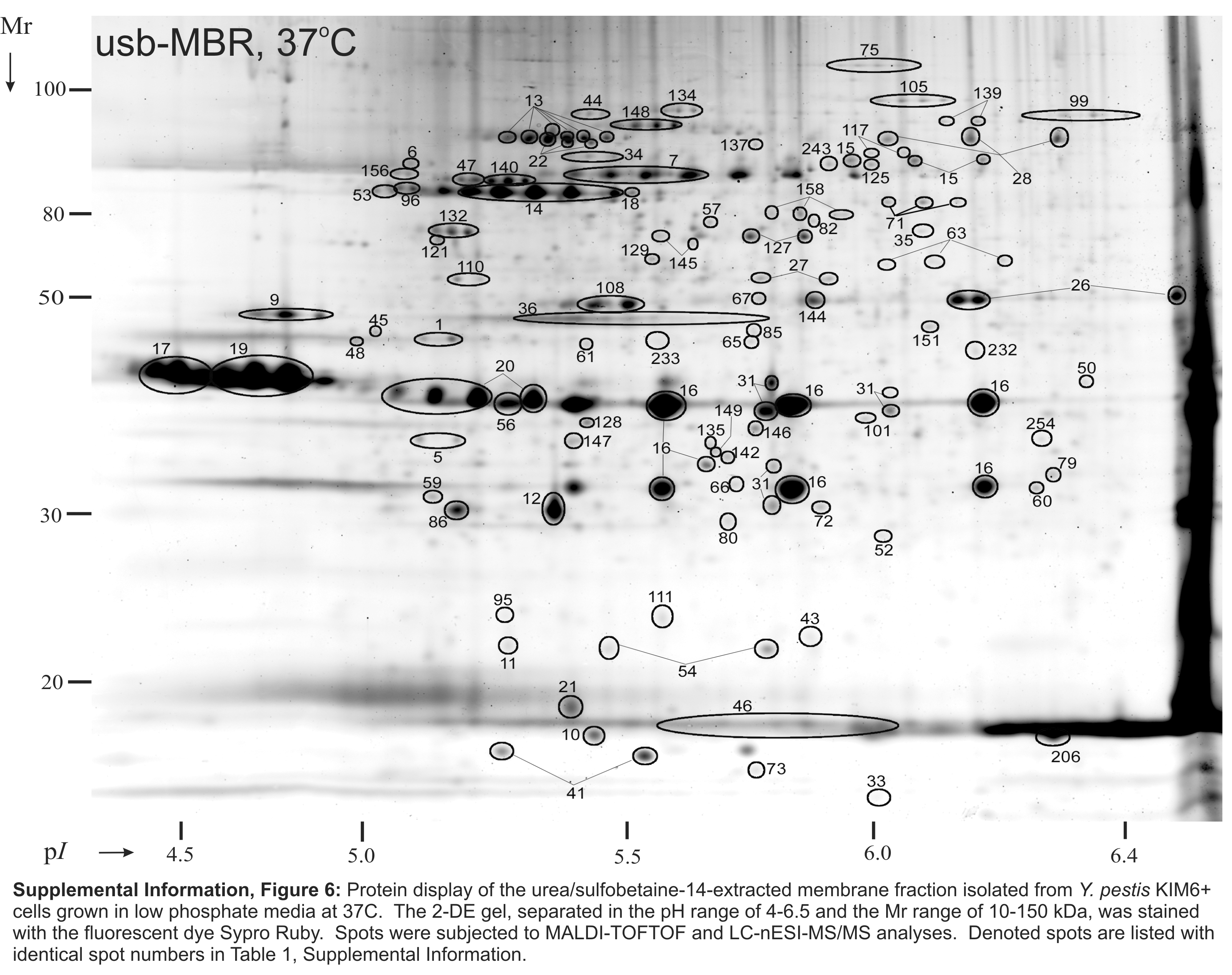

Supplement: Additional file 7 — Supplemental Information (Figure Six). Protein display of urea/sulfobetaine-14-extracted membrane fraction isolated from Y. pestis KIM6+ cells grown in low phosphate media at 37°C (pH range of 4–6.5). [file 1477-5956-7-5-S7.tiff]

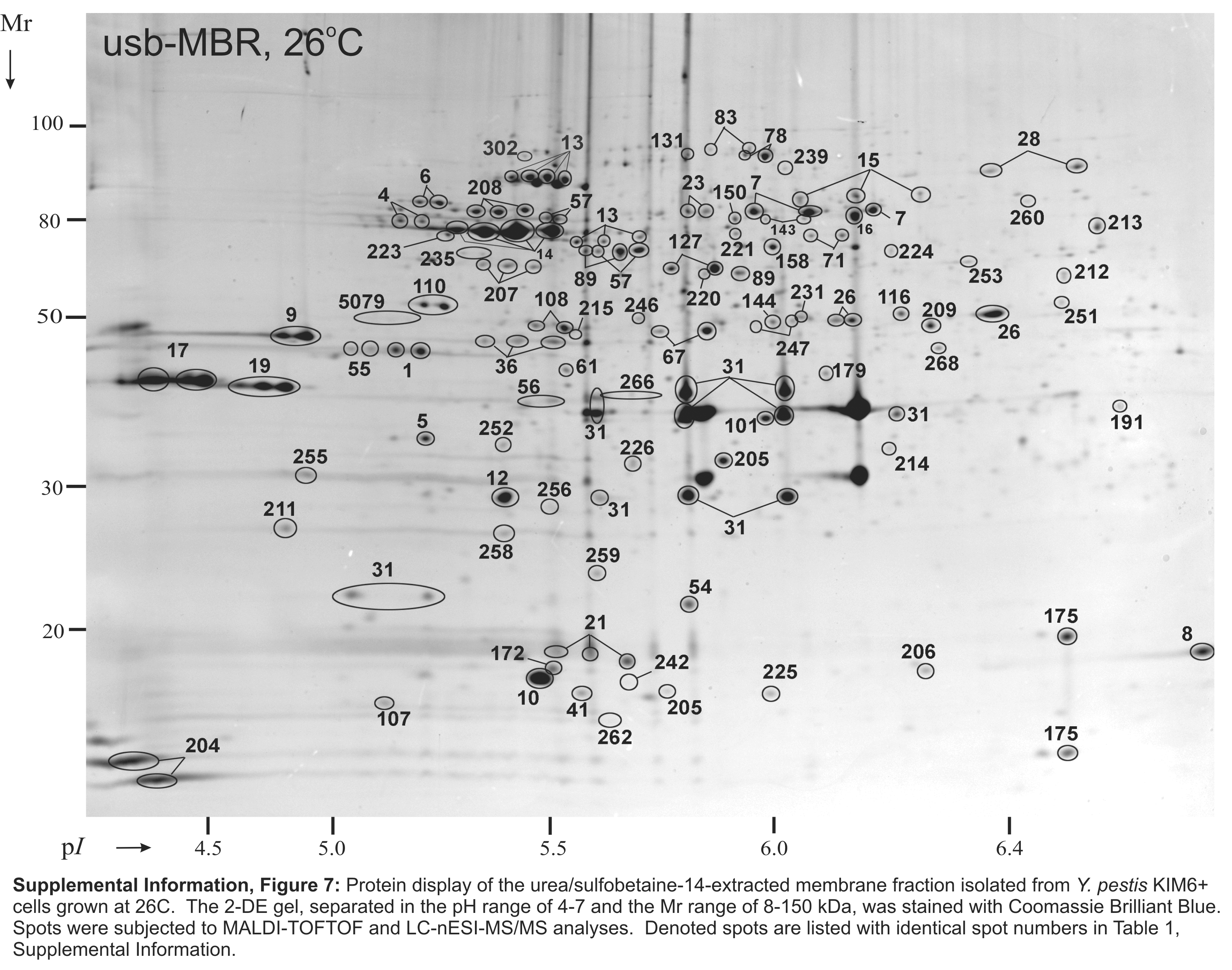

Supplement: Additional file 8 — Supplemental Information (Figure Seven). Protein display of urea/amidosulfobetaine-14-extracted membrane fraction isolated from Y. pestis KIM6+ cells grown to stationary phase at 26°C (pH range of 4–7). [file 1477-5956-7-5-S8.tiff]

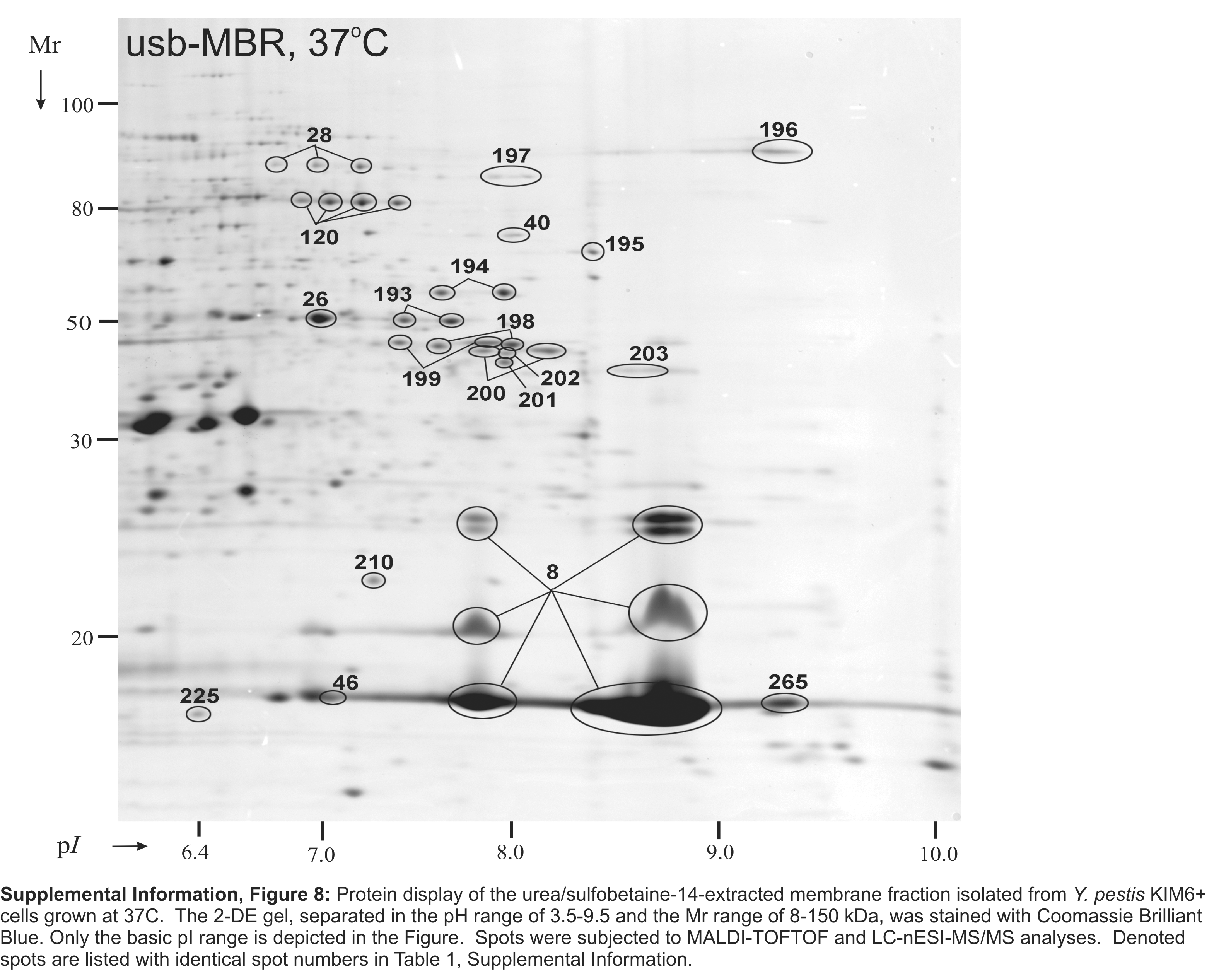

Supplement: Additional file 9 — Supplemental Information (Figure Eight). Protein display of urea/amidosulfobetaine-14-extracted membrane fraction isolated from Y. pestis KIM6+ cells grown at 37°C (pH range of 6–10). [file 1477-5956-7-5-S9.tiff]

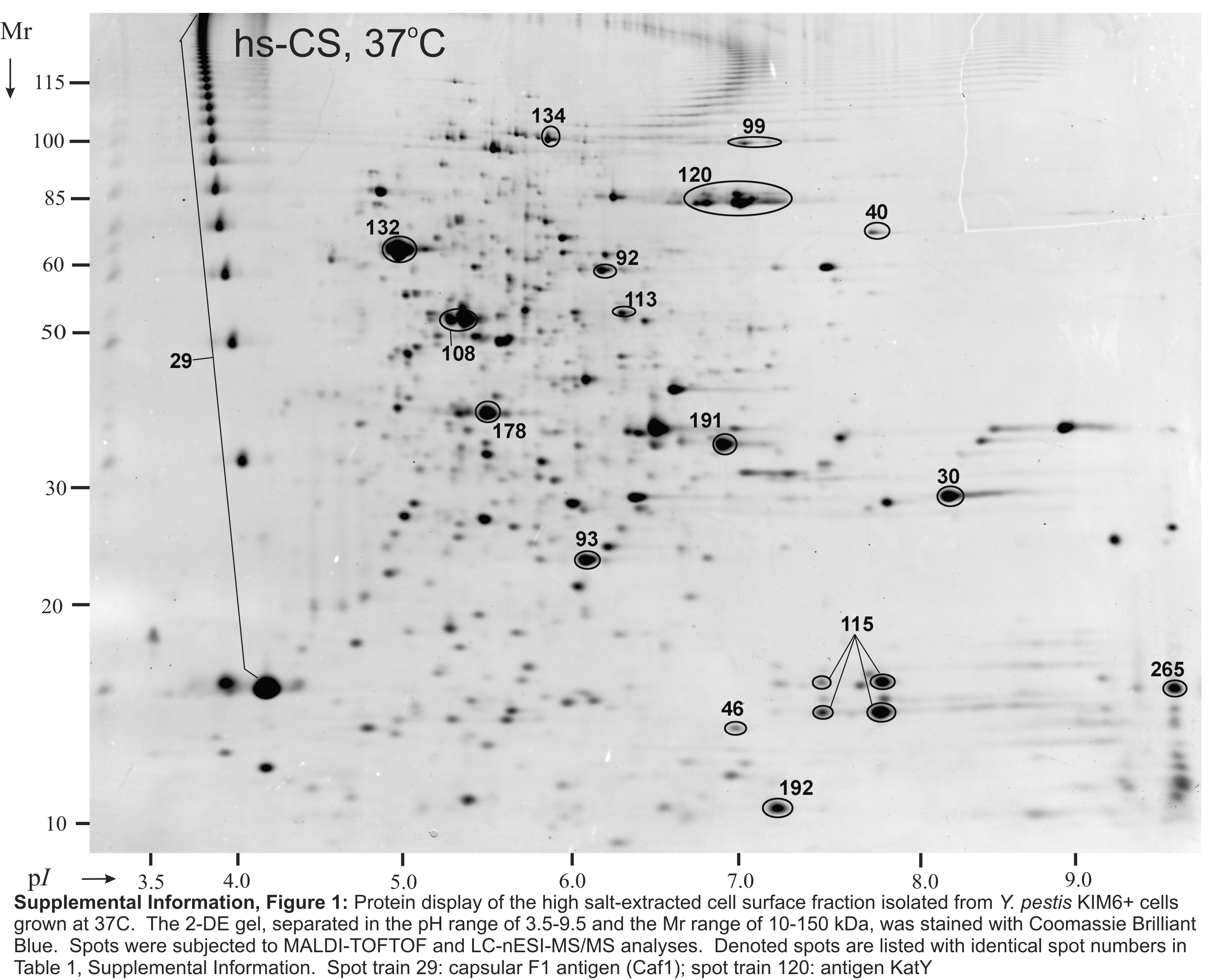

Supplement: Additional file 10 — Supplemental Information (Figure One). Protein display of the high salt-extracted cell surface fraction isolated from Y. pestis KIM6+ cells grown at 37°C (pH range 3.5–10). [file 1477-5956-7-5-S10.tiff]
